# Emergent scale-free networks

**DOI:** 10.1093/pnasnexus/pgae236

**Published:** 2024-06-14

**Authors:** Christopher W Lynn, Caroline M Holmes, Stephanie E Palmer

**Affiliations:** Department of Physics, Yale University, New Haven, CT 06511, USA; Quantitative Biology Institute, Yale University, New Haven, CT 06511, USA; Wu Tsai Institute, Yale University, New Haven, CT 06510, USA; Initiative for the Theoretical Sciences, Graduate Center, City University of New York, New York, NY 10016, USA; Department of Physics, Princeton University, Princeton, NJ 08544, USA; Department of Physics, Princeton University, Princeton, NJ 08544, USA; Department of Organismic and Evolutionary Biology, Harvard University, Cambridge, MA 02138, USA; Department of Organismal Biology and Anatomy, University of Chicago, Chicago, IL 60637, USA; Department of Physics, University of Chicago, Chicago, IL 60637, USA

**Keywords:** complex networks, scale-free structure, self-organization, network science

## Abstract

Many complex systems—from the Internet to social, biological, and communication networks—are thought to exhibit scale-free structure. However, prevailing explanations require that networks grow over time, an assumption that fails in some real-world settings. Here, we explain how scale-free structure can emerge without growth through network self-organization. Beginning with an arbitrary network, we allow connections to detach from random nodes and then reconnect under a mixture of preferential and random attachment. While the numbers of nodes and edges remain fixed, the degree distribution evolves toward a power-law with an exponent $\gamma =1+\frac{1}{p}$ that depends only on the proportion *p* of preferential (rather than random) attachment. Applying our model to several real networks, we infer *p* directly from data and predict the relationship between network size and degree heterogeneity. Together, these results establish how scale-free structure can arise in networks of constant size and density, with broad implications for the structure and function of complex systems.

**Significance Statement**

Scale-free structure is foundational to our understanding of complex networks. Yet, while most explanations require networks to constantly grow, many real-world systems—from the wiring of the brain to protein interactions and ecological relationships—fluctuate around a constant size. This raises a clear question: How can scale-free structure emerge without growth? Here, we propose a minimal model in which the numbers of nodes and edges remain constant, but scale-free structure arises through the self-organization of connections. Despite its simplicity, the model quantitatively describes an array of real-world networks, demonstrating how scale-free structure can emerge in systems of constant size and density.

## Introduction

Scale-free structure is a hallmark feature of many complex networks, with the probability of a node having *k* links (or degree *k*) following a power law $k^{-\gamma }$. First studied in networks of scientific citations (1, 2), scale-free structure has now been reported across a wide array of complex systems, from social networks [of romantic relationships (3), scientific collaborations (4), and online friendships (5)]; to biological networks [of connections in the brain (6), metabolic interactions (7), and food webs (8, 9)]; to the online and physical wiring of the Internet (10–13); to language (14), transportation (15), and communication networks (16). Although empirically measuring power laws in real networks poses important technical challenges (17, 18), the study of scale-free structure continues to provide deep insights into the nature of complex systems (19–28).

Existing explanations for scale-free structure primarily rely on two mechanisms: preferential attachment (such that well-connected nodes are more likely to gain new connections) and growth (wherein nodes are constantly added to the network) (2, 21). While alternatives have been proposed to preferential attachment [such as random attachment to edges (29), random copying of neighbors (30), and deterministic attachment rules (31)], the dependence on growth remains widespread. In many real-world contexts, however, constant growth is unrealistic. For example, the number of neurons in the brain does not grow without bound (32), and just as animals are added to a population and words are added to a language, others die out. In these systems, network size and density fluctuate around steady-state values, and structural properties emerge without growth through the self-organization of nodes and edges. Thus, understanding whether, and how, scale-free structure emerges without growth remains a central open question.

## Emergent scale-free structure in real networks

**Fig. 1.**
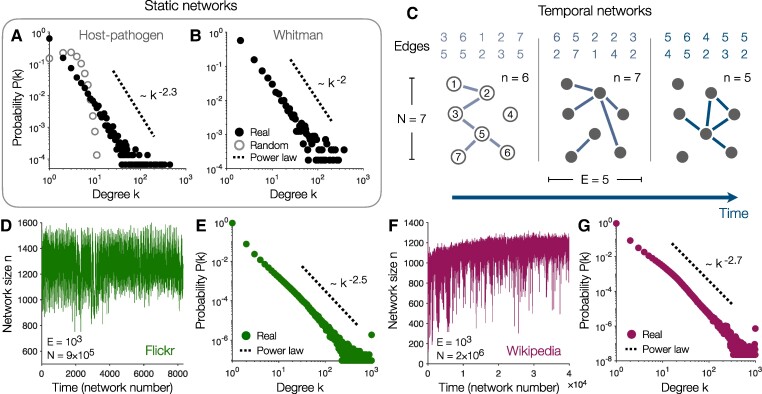
Degree distributions of real networks. A) Degree distribution of host–pathogen relationships between plant and animal species (33) and for a random network with the same numbers of nodes and edges. B) Degree distribution for the network of transitions between nouns in Walt Whitman’s *Leaves of Grass* (26). C) Procedure for analyzing temporal network dynamics. We divide the sequence of edges into groups of equal size *E*, thus forming a series of network snapshots. Each snapshot contains n≤N nodes, where *N* is the total number of nodes in the dataset. D) Trajectory of system size *n* over time for the network of friendships on Flickr (34). E) Degree distribution of the Flickr network computed across all network snapshots. F–G) Trajectory of network size F) and degree distribution G) for the hyperlinks between pages on English Wikipedia (35). Dashed lines illustrate power laws.

To understand the importance of network self-organization, we begin by empirically demonstrating that many real-world systems can maintain scale-free structure even without growth. In some complex systems—including many biological and language networks—topological properties (such as scale-free structure) arise without constant growth. For example, in a network of host–pathogen relationships between plant and animal species (33), we find that the degree distribution P(k) exhibits power-law scaling (Fig. 1A); and we observe similar scale-free structure in the transitions between words in the English language (Fig. 1B) (26). By contrast, if we randomize the connections between nodes, then the degrees drop off super-exponentially as a Poisson distribution, and the scale-free structure vanishes (Fig. 1A).

Meanwhile, other systems—particularly online social and communication networks, scientific collaborations, and the Internet—are often viewed as growing by accumulating new nodes and edges over time (19). But by studying their temporal dynamics, we will see that even these networks can exhibit scale-free structure without growth. The dynamics of a network are defined by a sequence of connections $\left(i_{t},j_{t}\right)$, ordered by the time *t* at which they occur. Letting these edges accumulate over time, we arrive at a single growing network. Alternatively, one can divide the connections into groups of equal size *E*, thus defining a sequence of independent snapshots, each representing the structure of the network within a specific window of time (Fig. 1C). For clarity, we let *N* denote the total number of nodes in the entire sequence, while *n* reflects the number of nodes in a single snapshot (Fig. 1C). Consider, for example, the social network of friendships on Flickr (Fig. 1D–E) (34). Dividing the sequence of connections into groups of size $E=10^{3}$, we can study the evolution of different network properties. In particular, we find that the Flickr network fluctuates around a constant size *n* (Fig. 1D). Yet even without growing, we see that the network maintains a clear power-law degree distribution (Fig. 1E), and we verify that this scale-free structure remains consistent over time (see Supplementary material).

We can repeat the above procedure for any time-evolving network, such as the links between pages on Wikipedia (Fig. 1F–G) (35). Across a number of different social, web, communication, and transportation networks (see Supplementary material), we divide the dynamics into snapshots with $E=10^{3}$ edges each, the largest number that can be applied to all systems. While some networks grow slowly in time (such as Wikipedia in Fig. 1F), all of the networks approach a steady-state size (see Supplementary material). In fact, the snapshots are limited to n≤2E by definition, and therefore cannot grow without bound. Even still, many of the networks exhibit scale-free structure (such as Flickr and Wikipedia in Fig. 1E,G). In what follows, we will develop a simple dynamical model capable of describing all of these networks.

## Model of emergent scale-free networks

**Fig. 2.**
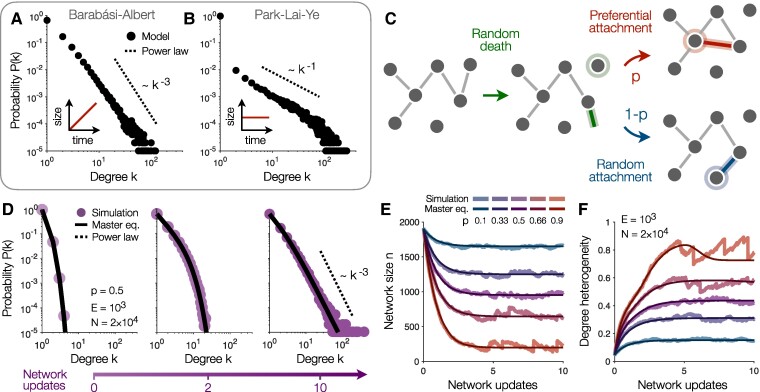
Modeling the emergence of scale-free structure. A–B) Degree distributions for the Barabábasi–Albert A) and Park–Lai–Ye B) models of scale-free networks (see Supplementary material for descriptions) (21, 45). C) Network dynamics in our model. Beginning with an arbitrary network (*left*), at each step in time a random node loses all of its connections (*center*). Each disconnected edge attaches to another node either preferentially (with probability *p*; *top right*) or randomly (with probability 1−p; *bottom right*). D) Degree distributions for initially random networks (*left*), after two full network updates (that is, after 2N steps of the dynamics; *center*), and after ten network updates (*right*) with $E=10^{3}$ edges, $N=2\times 10^{4}$ nodes (for average degree $\bar{k}=0.1$), and equal amounts of preferential and random attachment (p=0.5). Solid lines depict predictions from the master equation (Eq. 1), and dashed line illustrates a power law for comparison. E–F) Trajectories of network size *n* E) and degree heterogeneity F) over the course of ten network updates (10N steps of the dynamics) for different values of *p*. Thick lines reflect individual simulations (beginning with random networks), and thin lines represent master equation predictions. Distributions in panels A, B, and D are computed over 100 simulations (see Supplementary material).

The above results demonstrate that scale-free structure can arise without growth in real networks. Yet most existing models of scale-free networks (such as the Barabási–Albert model in Fig. 2A) rely on the addition of nodes and edges over time (19, 21). To address this gap, a large number of models have been proposed for scale-free networks without growth (36–47). For instance, power-law degree distributions can result from the optimization of network properties or by connecting nodes based on fitness (36, 37, 39, 43). In fact, one can reproduce any degree distribution by designing replacement nodes with a desired connectivity profile (41, 42). However, these models rely on global design choices for the optimization, fitness, or connectivity functions, rather than explaining the self-organization of network structure.

Here we present a minimal model in which a fixed number of nodes and edges rewire to produce power-law degree distributions with a wide range of exponents γ≥2. We begin with an arbitrary network of *N* nodes and *E* edges (for simplicity, we always begin with a random network). At each time step, one node is selected at random to lose all of its connections (Fig. 2C, *center*). Each of these connections then reattaches in one of two ways: (i) with probability *p*, it connects to a node via preferential attachment (that is, it attaches to node *i* with probability proportional to its degree $k_{i}$; Fig. 2C, *top right*), or (ii) with probability 1−p, it connects to a random node (Fig. 2C, *bottom right*). In this way, the total numbers of nodes *N* and edges *E* remain constant, with the wiring between nodes simply rearranging over time. Notably, besides *N* and *E*, the model only contains a single parameter *p*, representing the proportion of preferential (rather than random) attachment.

Previous models have considered similar mixtures of preferential and random attachment in growing networks (48). Meanwhile, many investigations have studied networks that simultaneously add and delete nodes and edges; yet to produce scale-free structure, most of these models require addition to outpace deletion such that the network still grows on average (38, 40, 44, 46). Finally, there exist preferential attachment models that do not require growth (45, 47), but these only produce a limited range of power-law exponents γ≤1 that do not apply to many real-world networks (Fig. 2B). But by combining node death, preferential attachment, and random attachment, can realistic scale-free structure emerge without growth?

To answer this question, we can write down a master equation describing the evolution of the degree distribution $P_{t}(k)$ from one time step *t* to the next. At each step, the detachment of a random node (Fig. 2C, *center*) yields an average decrease in probability of $-\frac{1}{N}P_{t}(k)$. On average, killing a node produces $\bar{k}=2E/N$ disconnected edges that must be reattached. With probability *p*, each edge attaches preferentially (Fig. 2C, *top right*), connecting to a node of degree *k* with probability $\frac{k}{2E}$; on average, this preferential attachment yields an increase in probability of $\bar{k}p\frac{k-1}{2E}P_{t}(k-1)$ and a decrease of $-\bar{k}p\frac{k}{2E}P_{t}(k)$. Alternatively, with probability 1−p, each disconnected edge reattaches randomly (Fig. 2C, *bottom right*), yielding an increase in probability of $\bar{k}(1-p)\frac{1}{N}P_{t}(k-1)$ and a decrease of $-\bar{k}(1-p)\frac{1}{N}P_{t}(k)$. Combining these contributions and simplifying, we arrive at the master equation,


(1) $$\begin{array}{rl} P_{t+1}(k) & =P_{t}(k)+\frac{1}{N}[-P_{t}(k)+p((k-1)P_{t}(k-1)-kP_{t}(k))\\ & \;+\bar{k}(1-p)(P_{t}(k-1)-P_{t}(k))]. \end{array}$$

We are now prepared to study the evolution of the degree distribution. To compare against the real temporal networks (for which N≳E), we begin by randomly placing $E=10^{3}$ edges among $N=2\times 10^{4}$ nodes, for an average degree $\bar{k}=0.1$. Running the dynamics with equal amounts of preferential and random attachment (such that p=0.5), we find that the master equation (Eq. 1) provides a close approximation to simulations (Fig. 2D). As the connections rearrange, the degree distribution, which is initially Poisson (Fig. 2D, *left*), quickly broadens (Fig. 2D, *center*). Eventually, the distribution develops a clear power law $P(k)∼k^{-\gamma }$ in the high-degree limit k≫1, with a realistic exponent γ=3 (Fig. 2D, *right*). We therefore find that realistic scale-free structure emerges without growth from our simple dynamics (Fig. 2C).

This self-organization of scale-free structure leaves an imprint on network properties beyond just the degree distribution. Consider, for example, the size of the network *n*, which (for consistency with the real networks) is defined as the number of nodes with at least one connection. As the dynamics unfold, edges tend to collect around a small number of high-degree hubs, thus decreasing the size of the network *n* (Fig. 2E). These hubs comprise the heavy tail of the degree distribution. To quantify this heavy tail, rather than using the variance of the degrees (which diverges for power-law distributions with γ≤3), we instead compute the *heterogeneity*  $\frac{1}{2}\langle |k_{i}-k_{j}|\rangle /\langle k\rangle$, which is normalized to lie between zero and one, where ⟨⋅⟩ represents an average over degrees k≥1 and $\left\langle |k_{i}-k_{j}|\right\rangle$ measures the average absolute difference in degrees (26). As the network evolves, and scale-free structure emerges (Fig. 2D), we see that the degree heterogeneity increases (Fig. 2F). Notably, both the network size and degree heterogeneity approach steady-state values, with larger proportions *p* of preferential attachment yielding networks that are smaller (Fig. 2E), yet more heterogeneous (Fig. 2F).

## Steady-state scale-free structure

Thus far, we have explored the network dynamics numerically (using the master equation) and through simulations. But the simplicity of our model gives us the opportunity to solve for the steady-state degree distribution analytically. Setting $P_{t}(k)=P_{t+1}(k)=P(k)$, the master equation reduces to the recursion relation


(2) $$P(k)=\frac{\;p(k-1)+\bar{k}(1-p)}{1+pk+\bar{k}(1-p)}P(k-1).$$

In the thermodynamic limit N,E→∞ (holding fixed the average degree $\bar{k}=2E/N$), one can then solve for the steady-state distribution


(3) $$P(k)=\frac{1}{C}\frac{\Gamma \left(k+\bar{k}\left(\frac{1}{\;p}-1\right)\right)}{\Gamma \left(k+\bar{k}\left(\frac{1}{\;p}-1\right)+1+\frac{1}{\;p}\right)},$$

where *C* is the normalization constant and Γ(⋅) is Euler’s gamma function. In what follows, we normalize P(k) to run over positive degrees k≥1, such that


(4) $$C=\frac{\Gamma \left(\frac{1}{\;p}\right)\Gamma \left(1+\bar{k}\left(\frac{1}{\;p}-1\right)\right)}{\Gamma \left(1+\frac{1}{\;p}\right)\Gamma \left(1+\frac{1}{\;p}+\bar{k}\left(\frac{1}{\;p}-1\right)\right)}.$$

In the high-degree limit $k\gg \bar{k}/p$, the above distribution falls off as a power law $P(k)∼k^{-\gamma }$ with scale-free exponent $\gamma =1+\frac{1}{p}$ (see Supplementary material). We therefore find that the network dynamics produce a wide range of exponents γ≥2 observed in real-world systems. Moreover, this scale-free structure depends only on the proportion *p* of preferential attachment (independent from the average degree $\bar{k}$).

**Fig. 3.**
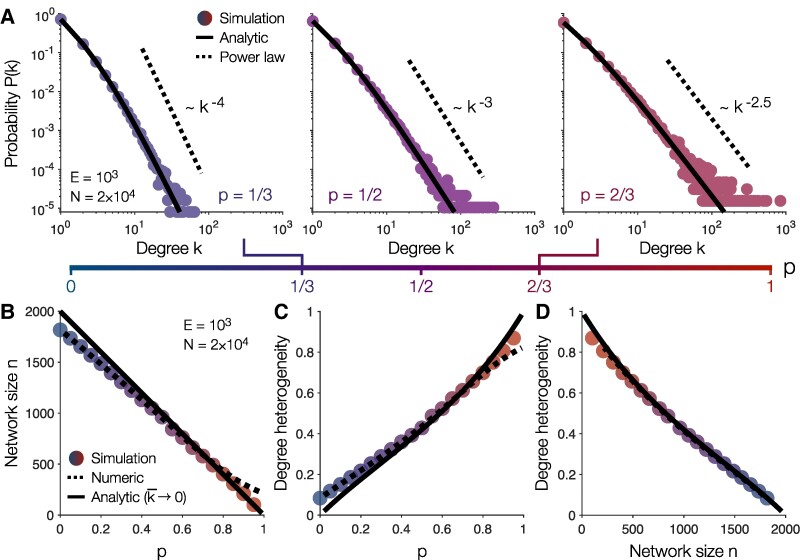
Analytic predictions in steady-state. A) Steady-state degree distributions for increasing proportions *p* of preferential attachment in networks with $E=10^{3}$ edges and $N=2\times 10^{4}$ nodes (for average degree $\bar{k}=0.1$). Data points depict simulations (see Supplementary material), solid lines reflect the analytic prediction in Eq. 3, and dashed lines illustrate power laws with the predicted exponents $\gamma =1+\frac{1}{p}$. B–C) Network size *n* B) and degree heterogeneity C) as functions of *p*. D) Degree heterogeneity versus network size while sweeping over *p*. In panels B–D, data points are computed using simulations, dashed lines are calculated numerically using Eq. 3, and solid lines are analytic predictions in the limit of sparse connectivity $\bar{k}\to 0$ (see Supplementary material).

We confirm the analytic distribution (Eq. 3) and the power-law tail in simulations (Fig. 3A). For equal amounts of preferential and random attachment (p=0.5), the model generates a scale-free exponent γ=3 (Fig. 3A, *center*), as observed previously in Fig. 2D. For larger proportions *p* of preferential attachment, high-degree hubs become more prevalent, strengthening the heavy tail in P(k) and decreasing the exponent *γ* (Fig. 3A, *right*). Indeed, as *p* increases, the dynamics produce networks that are smaller (Fig. 3B) and more heterogeneous (Fig. 3C; see Supplementary material for analytic predictions). Our model thus predicts a specific inverse relationship between network size and heterogeneity (Fig. 3D), which we will be able to test in real networks. Together, these results establish analytically that our simple network dynamics give rise to scale-free structure with realistic exponents *γ*.

## Modeling real networks

**Fig. 4.**
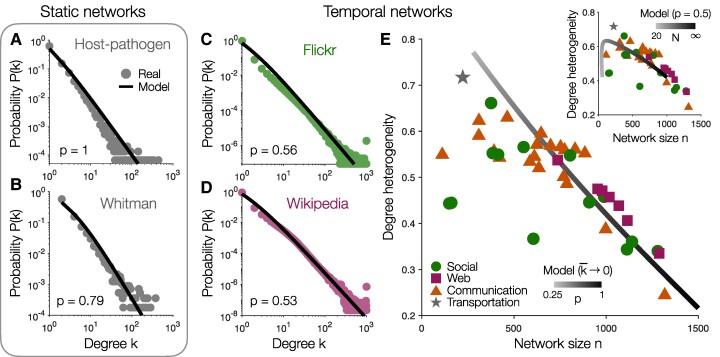
Comparing real and model networks. A–B) Degree distributions for static networks of host–pathogen relationships A) and word transitions B) (26, 33). Solid lines reflect analytic predictions of our model (Eq. 3) with $\bar{k}=2E/N$ set to match the average degree in each network and *p* fit to the observed degree distribution (see Supplementary material). C–D) Degree distributions for temporal networks of friendships on Flickr C) and hyperlinks on English Wikipedia D) (34, 35) compared to analytic model predictions (solid lines) with $E=10^{3}$, *N* set to the number of nodes in each dataset, and *p* fit to the observed degree distributions. E) Degree heterogeneity as a function of network size *n* across the temporal networks listed in the Supplementary material, where each data point represents an average over snapshots. Lines reflect numeric model predictions while sweeping over *p* (with $\bar{k}\to 0$) or sweeping over the number of nodes *N* (with p=0.5; *inset*).

Ultimately, we would like to use our model to study real-world systems. To compare against static networks (such as those in Fig. 1A–B), we can set $\bar{k}$ to match the average degree in the observed system. This leaves one free parameter, the proportion *p* of preferential attachment, which we can fit to the degree distribution of a given network (see Supplementary material). For example, the host–pathogen network is best described as arising entirely from preferential attachment (such that p=1; Fig. 4A), while the transitions between words reflect 80% preferential attachment (Fig. 4B). To analyze temporal networks (such as those in Fig. 1C–G, we fix the number of edges (here $E=10^{3}$) and set the number of nodes in the model *N* to be the total number that appear in a given dataset. Fitting *p* to each degree distribution, we find the Flickr and Wikipedia networks are best described as arising from nearly equal amounts of preferential and random attachment (such that p≈0.5; Fig. 4C–D). In fact, despite only fitting one parameter, our simple model provides a surprisingly good description across a wide range of static and temporal networks (see Supplementary material).

As we sweep over *p*, adjusting the ratio of preferential to random attachment, the model predicts a specific tradeoff between the size of a network and its degree heterogeneity (Fig. 3D). Computing the average properties (over different snapshots) for each of the temporal networks, we find a similar inverse relationship between network size and heterogeneity (Fig. 4E). If we instead hold *p* fixed and sweep over the number of nodes *N*, the model also predicts the drop in degree heterogeneity observed in small networks (Fig. 4E, *inset*). Moreover, even at the level of individual networks, we discover similar tradeoffs between size and heterogeneity across different snapshots (see Supplementary material). We therefore find that our model not only captures the degree distributions observed in real-world systems (Fig. 4A–D; Supplementary material), but it also predicts the relationships between different network properties (Fig. 4E).

## Extensions and robustness

**Fig. 5.**
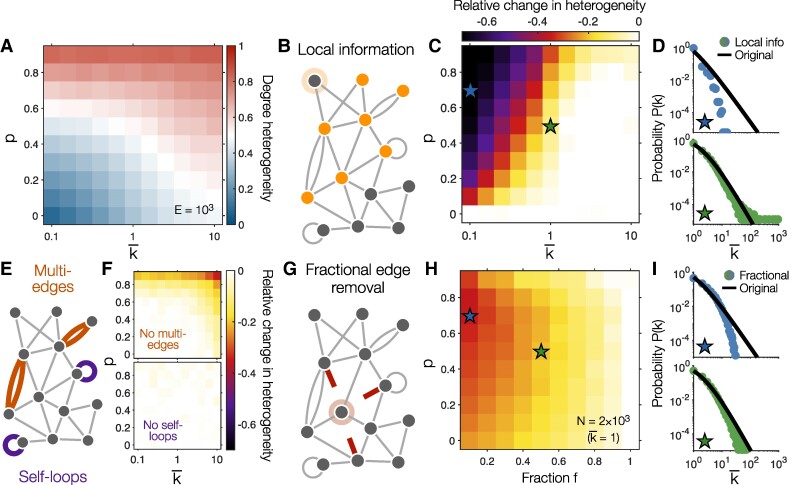
Extending the original model. A) Degree heterogeneity of the original model (Fig. 2C) while sweeping over the preferential attachment proportion *p* and average degree $\bar{k}$ for networks with $E=10^{3}$ edges. B) Constraining to local information, each node can preferentially attach only to its neighbors and their neighbors. C) Relative change in degree heterogeneity after restricting to local information while sweeping over *p* and $\bar{k}$. D) Degree distributions for the original model (Eq. 3; solid lines) and with local information (data points) for parameters *p* and $\bar{k}$ indicated in panel B. E) Multiedges and self-loops are allowed in the original model. F) Relative change in heterogeneity when disallowing multiedges (*top*) or self-loops (*bottom*). G) When a node detaches from the network, rather than removing all of its edges, one could remove only a fraction *f*. H) Relative change in heterogeneity with fractional edge removal while sweeping over *p* and *f* (for networks of average degree $\bar{k}=1$). I) Degree distributions for the original model (Eq. 3; solid lines) and with fractional edge removal (data points) for parameters *p* and *f* indicated in panel H. In all panels, values are computed using simulated networks with $E=10^{3}$ edges (see Supplementary material).

In designing the model (Fig. 2C), we sought the simplest dynamics that would self-organize to produce scale-free structure. Given this simplicity, there are a number of natural extensions one could explore. To investigate the impact of model extensions on the degree distribution P(k), we again consider the heterogeneity of degrees. In the original model (with the number of edges *E* held fixed), as we sweep over the proportion *p* of preferential attachment and the number of nodes *N* (or, equivalently, the average degree $\bar{k}=2E/N$), we arrive at a phase diagram for the network structure (Fig. 5A). As *p* and $\bar{k}$ increase, the network dynamics produce degree distributions with heavier tails, thus increasing the degree heterogeneity (Fig. 5A).

When performing preferential attachment, we note that these simple dynamics (Fig. 2C) rely on global information about the degrees of all the nodes in a network. In some scenarios, however, a node may only have access to local information about the degrees of nodes in its own neighborhood (for example, its neighbors and their neighbors; Fig. 5B) (49, 50). Restricting to local information, we find that the degree heterogeneity is significantly reduced for large *p* (when preferential attachment dominates) and small $\bar{k}$ (when connections are sparse, and therefore local information becomes severely restrictive; Fig. 5C–D, *top*). By contrast, for $\bar{k}≳1$, the networks are dense enough that local information is sufficient to generate heterogeneous degrees (Fig. 5C) and, indeed, scale-free structure (Fig. 5D, *bottom*).

Beyond global information, the original model also allows multiedges (where two nodes are connected by multiple edges; Fig. 5E, *top*) and self-loops (where a node connects to itself; Fig. 5E, *bottom*). If we disallow multiedges, the network dynamics still produce scale-free structure for all of parameter space besides p≥0.9 and $\bar{k}\gg 1$ (when networks are both highly heterogeneous and dense; Fig. 5F, *top*). Similarly, if we disallow self-loops, the degree distribution is almost entirely unaffected (Fig. 5F, *bottom*). As a final extension, when a node detaches from the network, rather than losing all of its connections, one could imagine that it only loses some fraction *f* (Fig. 5G). In the limit f=0, the dynamics halt and the network becomes static. As *f* increases, so too does the degree heterogeneity, until at f=1, we recover the original model (Fig. 5H). Indeed, as long as dying nodes lose a fraction f≳0.5 of their edges, the model still produces power-law degree distributions (Fig. 5I, *bottom*), which we confirm for different average degrees $\bar{k}$ (see Supplementary material). In combination, these results demonstrate specific ways that the network dynamics can be extended, restricted, and generalized, while still giving rise to scale-free structure.

## Conclusion

Understanding how scale-free structure arises from fine-scale mechanisms is central to the study of complex systems. However, existing mechanisms typically require constant growth, an assumption that fails in many real-world networks. Even in networks that are usually viewed as growing, we show that individual snapshots (which cannot grow without bound by definition) can still exhibit scale-free structure (Fig. 1). Here, we propose a minimal model in which scale-free structure emerges naturally through the self-organization of nodes and edges. By allowing nodes to detach from the network, and letting connections rearrange under a mixture of preferential and random attachment, we show (both analytically and through simulations) that the degree distribution develops a power-law tail $P(k)∼k^{-\gamma }$ (Fig. 2). Moreover, the scale-free exponent (which takes the simple form $\gamma =1+\frac{1}{p}$) only depends on the proportion *p* of preferential attachment and captures a wide range of values γ≥2 observed in real systems (Fig. 3). In fact, despite containing only one free parameter, the model provides a surprisingly good description of many real-world networks (Fig. 4; Supplementary material).

However, due to the simplicity of the model, it does not have the flexibility to incorporate all of the processes observed in real network dynamics. By design, the numbers of nodes and edges remain fixed, and so our model may not apply to growing networks for which snapshots are not available. Similarly, because our model is based on node death and edge rearrangement (Fig. 2C), we do not address the birth and death of edges studied previously (40). Finally, in its current form our model only includes undirected edges, and thus does not apply to directed networks.

Despite these limitations, the simplicity of our model also means that it can be easily extended to include additional features and mechanisms. For example, here we investigate the effects of local information, multiedges, self-loops, and fractional edge removal (Fig. 5). Future work can build upon this progress to develop new models for the emergence of scale-free networks. Beyond node degrees, we note that power-law distributions also arise in many other contexts, from the strengths of connections in the brain and the frequencies of words in language to the populations of cities and the net worths of individuals (18, 51). Do these power laws rely on the constant growth of a system? Or, instead, can scale-free distributions arise through the self-organization of existing resources? The framework presented here may provide fundamental insights to these questions.

## Supplementary Material

pgae236_Supplementary_Data

## Data Availability

The data analyzed in this paper and the code used to perform the analyses are openly available at: github.com/ChrisWLynn/Emergent{_}scale{_}free.

## Preprints

A preprint of this article is published at: arxiv.org/abs/2210.06453.
