# Supplementary material for: Emergent scale-free networks
Source: PNAS Nexus. 2024 Jun 14;3(7):pgae236. doi: 10.1093/pnasnexus/pgae236 (PMC11223655; doi:10.1093/pnasnexus/pgae236)
Supplement: pgae236_Supplementary_Data [file pgae236_supplementary_data.pdf]

Supplementary Materials

Emergent scale–free networks

Christopher W. Lynn[1,2,3,4,5], Caroline M. Holmes[5,6], & Stephanie E. Palmer[7,8]

[1] *Department of Physics, Yale University, New Haven, CT 06511, USA*

[2] *Quantitative Biology Institute, Yale University, New Haven, CT 06511, USA*

[3] *Wu Tsai Institute, Yale University, New Haven, CT 06510, USA*

[4] *Initiative for the Theoretical Sciences, Graduate Center, City University of New York, New York, NY 10016, USA*

[5] *Department of Physics, Princeton University, Princeton, NJ 08544, USA*

[6] *Department of Organismic & Evolutionary Biology, Harvard University, Cambridge, MA 02138, USA*

[7] *Department of Organismal Biology & Anatomy, University of Chicago, Chicago, IL 60637, USA*

[8] *Department of Physics, University of Chicago, Chicago, IL 60637, USA*

Contents

1 Introduction 3

2 Materials and methods 3

3 Consistency of scale–free structure over time 7

4 Temporal network datasets 8

5 Dynamics and degree distributions for temporal networks 10

6 Comparison with existing models of scale–free structure 15

7 Deviations between real and model networks 17

8 Properties of temporal network snapshots 19

9 Static networks 21

10 Fractional edge removal 24

References 25

1 Introduction

In this Supplementary Material, we provide extended analysis and discussion to support the results presented in the main text. The sections are ordered to align with their references in the main text. In Sec. 2, we present the materials and methods needed to reproduce the analytical and computational results in the main text. In Sec. 3, we investigate the temporal evolution of the Flickr network, showing that the degree distribution maintains a consistent power–law form over time. In Sec. 4, we list and describe the real temporal networks analyzed in the main text. In Sec. 5, for each of the temporal networks, we plot the evolution over time of the network size n as well as the degree distribution $P(k)$ (along with model fits). In Sec. 6, we compare our framework with existing models of scale–free structure. In Sec. 7, we examine a number of temporal networks that initially appear to be poorly described by our model; however, after fitting both the preferential attachment proportion p and the average degree $\bar{k}$, we show that our model provides a close fit to the observed degree distributions. In Sec. 8, we study the properties of individual network snapshots. In Sec. 9, we analyze an array of static networks, demonstrating that our model can be used to investigate networks without temporal data. Finally, in Sec. 10, we provide additional analysis of the model extension with fractional edge removal (Fig. 5G-I in the main text).

2 Materials and methods

2.1 Analyzing real–world networks

We analyze 41 different temporal networks (see Table 1) and 23 different static networks (see Table 2). Each network was selected to have a heavy–tailed (but not necessarily scale–free) degree distribution. The temporal networks fall into four distinct categories: social (reflecting the connections between people), web (representing hyperlinks between websites or the physical wiring of the Internet), communication (comprised of messages between people), and transportation (mapping flights between cities). All of the networks analyzed in this paper have been made openly available.

Each temporal network consists of a list of edges (i_t, j_t) with time stamps t. For every

such network, we denote the total number of nodes by N and the total number of edges (or the length of the dataset) by T. When examining the temporal dynamics, we divide the edges (i_t, j_t) into groups of size $E = 10^3$, the largest number that could be applied to all networks. Grouping the edges this way results in a sequence of $\lfloor T/E \rfloor$ network snapshots. Averaging over these snapshots, we can compute quantities such as the network size n (or the average number of nodes in each snapshot) and the degree heterogeneity.

To compute the degree distribution $P(k)$ for a given temporal network, we count the number of nodes of degree k across all snapshots. By definition, each degree distribution is normalized to run over all positive degrees $k \geq 1$. To estimate the proportion of preferential attachment p that best describes a given network, we fit the analytic distribution in Eq. S2 to the network's measured degree distribution. To begin, for static networks we set $\bar{k}$ to match the average degree in the system. For temporal networks, because each snapshot contains $E = 10^3$ edges (by definition), the average degree in our model is given by $\bar{k} = 2E/N$. We note that $\bar{k}$ differs from the total average degree $2T/N$ of the entire dataset. This leaves one free parameter p, which we compute by minimizing the root–mean–square error of the log probabilities. The degree distributions and model fits for all of the networks are displayed and discussed in the Supplemental Material. The gradient descent algorithm used to compute p has been made openly available.

2.2 Simulating network dynamics

In each simulation of our model, we begin with $E = 10^3$ edges (to match the real temporal networks) placed randomly among N nodes (allowing multi–edges and self–loops) and a preferential attachment proportion p. Since the temporal networks tend to have average degrees $\bar{k} \lesssim 1$ (Fig. S2C), we consider $N = 2 \times 10^4$ nodes for an average degree $\bar{k} = 0.1$ (unless otherwise specified). When simulating the dynamics, each step consists of one pass through the update rules. Specifically, we first select a random node and remove all of its connections. Each disconnected edge is then reattached in one of two ways: (i) with probability p, the edge attaches preferentially (that is, to node i with probability $\frac{k_i}{2E}$), or (ii) with probability $1 - p$, the

edge attaches randomly (that is, to a node selected uniformly at random).

After repeating this process N times, each node has detached from the network once (on average), which we refer to as one network update. Simulating our model therefore requires $O(mN)$ computations, where m is the number of network updates. Beginning with a random initialization of nodes and edges, we find that the networks typically reach their steady-state structures within $m = 10$ network updates. When computing steady–state properties, we first perform $m = 50$ network updates of burn–in followed by $m = 100$ network samples, each interspersed by one network update. Notably, simulations of our model scale linearly in N, thus allowing efficient investigations of large-scale networks.

In the main text, we investigate a number of model extensions, each of which can be implemented with minor changes to the above dynamics. To restrict to local information, when performing preferential attachment, each node is only allowed to connect to its neighbors and their neighbors. To remove multi–edges, when performing preferential or random attachment, nodes are disallowed from connecting to their neighbors. Similarly, to remove self–loops, nodes are disallowed from connecting to themselves. Finally, to implement fractional edge removal, when a node detaches from the network, it only loses a specified fraction f of its connections.

2.3 Steady–state predictions

Here, we derive a number of analytical results regarding the steady–state properties of our model. We begin with the master equation,

$$P_{t+1}(k) = P_t(k)+\frac{1}{N}\Big[-P_t(k)+p\Big((k-1)P_t(k-1)-kP_t(k)\Big)+\bar{k}(1-p)\Big(P_t(k-1)-P_t(k)\Big)\Big]. \quad \text{(S1)}$$

As described in the main text, in the thermodynamic limit $N, E \to \infty$ with the average degree $\bar{k} = 2E/N$ held fixed, one can derive solve for the steady–state degree distribution,

$$P(k) = \frac{1}{C}\frac{\Gamma\big(k+\bar{k}(\frac{1}{p}-1)\big)}{\Gamma\big(k+\bar{k}(\frac{1}{p}-1)+1+\frac{1}{p}\big)}, \quad \text{(S2)}$$

where C is the normalization constant and $\Gamma(\cdot)$ is Euler's gamma function. We normalize $P(k)$ to run over positive degrees $k \geq 1$, such that

$$C = \frac{\Gamma(\frac{1}{p})\Gamma(1+\bar{k}(\frac{1}{p}-1))}{\Gamma(1+\frac{1}{p})\Gamma(1+\frac{1}{p}+\bar{k}(\frac{1}{p}-1))}. \tag{S3}$$

Euler's gamma functions of the form $\Gamma(x+c)$ diverge as x^c in the limit $x \gg c$. Thus, in the high–degree limit $k \gg \bar{k}/p$, the degree distribution (Eq. S2) falls off as a power law,

$$P(k) \propto \frac{\Gamma\big(k+\bar{k}(\frac{1}{p}-1)\big)}{\Gamma\big(k+\bar{k}(\frac{1}{p}-1)+1+\frac{1}{p}\big)} \tag{S4}$$

$$\sim \frac{k^{\bar{k}(\frac{1}{p}-1)}}{k^{\bar{k}(\frac{1}{p}-1)+1+\frac{1}{p}}} \tag{S5}$$

$$= k^{-(1+\frac{1}{p})}. \tag{S6}$$

We therefore find that the degree distribution has a power–law tail $P(k) \sim k^{-\gamma}$ with scale–free exponent $\gamma = 1 + \frac{1}{p}$. We note that γ only depends on the proportion p of preferential attachment (independent of the average degree $\bar{k}$), and can achieve any value $\gamma \geq 2$.

Using the degree distribution (Eq. S2), we can derive analytic predictions for different network properties. For example, we note that the network size n (that is, the number of nodes with degree $k \geq 1$) is given simply by $n = N(1 - P(0))$, where $P(0)$ is the probability of a node having no connections. Throughout the paper, we normalize $P(k)$ to run over the positive degrees $k \geq 1$, but if we allow it to run over all degrees $k \geq 0$, then the normalization constant takes the form

$$C = \frac{\Gamma(\frac{1}{p})\Gamma(\bar{k}(\frac{1}{p}-1))}{\Gamma(1+\frac{1}{p})\Gamma(\frac{1}{p}+\bar{k}(\frac{1}{p}-1))}. \tag{S7}$$

Solving for the zero–degree probability $P(0) = (1 + \bar{k}(1-p))^{-1}$, we arrive at an analytic prediction for the network size,

$$n = \frac{N\bar{k}(1-p)}{1+\bar{k}(1-p)} = \frac{2E(1-p)}{1+\bar{k}(1-p)}. \tag{S8}$$

Thus, in the limit of sparse connectivity $\bar{k} \to 0$, we find that $n = 2E(1-p)$.

Let's now consider the degree heterogeneity $\frac{1}{2}\langle|k_i - k_j|\rangle / \langle k \rangle$, where $\langle\cdot\rangle$ indicates an average over positive degrees $k \geq 1$. Using $P(k)$, one can compute $\langle k \rangle = \bar{k} + \frac{1}{1-p}$, which

diverges as $p \to 1$ (and as $\gamma \to 2$). In the sparse connectivity limit $\bar{k} \to 0$, we can compute the average absolute difference in degrees $\langle |k_i - k_j| \rangle$ in a similar fashion, resulting in the following expression for the degree heterogeneity,

$$H = \frac{p}{1+p}\, {}_3F_2\Big(1, 1, 2;\ 1 + \frac{1}{p}, 2 + \frac{1}{p};\ 1\Big), \tag{S9}$$

where ${}_rF_q(\cdot)$ is the generalized hypergeometric function. In the limit of purely random dynamics ($p \to 0$), we see that the heterogeneity vanishes. Conversely, in the limit of purely preferential attachment ($p \to 1$), we have ${}_3F_2(1, 1, 2;\ 2, 3;\ 1) = 2$, and so the heterogeneity reaches its maximum possible value of one.

3 Consistency of scale–free structure over time

In the main text, we begin by investigating the temporal evolution of real–world networks (Fig. 1C-G in the main text). Each temporal network dataset is defined by a sequence of edges (i_t, j_t) ordered by the time t at which they occur. Dividing these edges into groups of size E (here, we consider $E = 10^3$), we arrive at a series of network snapshots. The number of nodes n in a given snapshot defines the size of the network at a specific moment in time. In the network of friendships on Flickr, for example, we see that the snapshots fluctuate around a constant size n (Fig. S1A).

In addition to the network size n, we can also study the evolution of the degree distribution over time. Computing $P(k)$ at three different points in the dataset (indicated in Fig. S1A), we find that the degree distribution maintains a consistent power–law form (Fig. S1B). Indeed, computing $P(k)$ across the entire dataset, we observe the same scale–free structure (Fig. S1C). Instead, if we randomize the structure of each snapshot, the degrees drop off super–exponentially as a Poisson distribution (Fig. S1C). Together, these results demonstrate that the Flickr network not only exhibits scale–free structure across different points in the dataset, but that the particular power–law form of the degree distribution remains consistent over time (Fig. S1B).

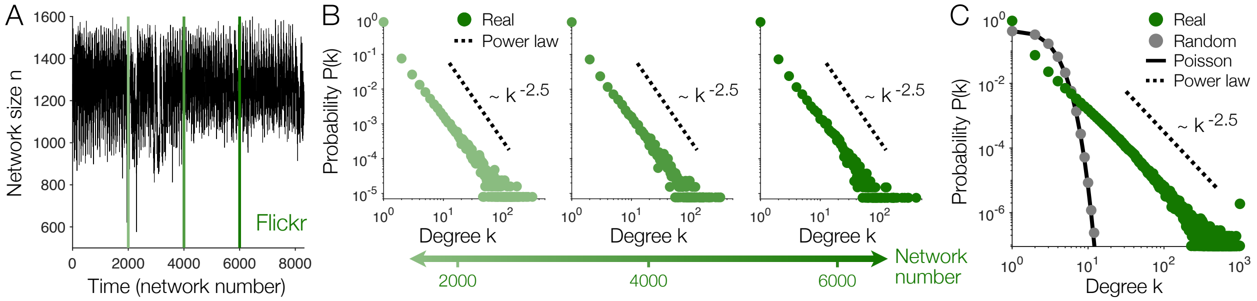

Fig. S1. Temporal evolution of Flickr network. (*A*) Trajectory of network size n over time. (*B*) Degree distributions $P(k)$ around the 2000th, 4000th, and 6000th snapshots, as illustrated in panel (*A*). Each distribution is computed over 100 consecutive snapshots. (*C*) Degree distribution computed across all snapshots (green) and after randomizing each snapshot (that is, generating a random snapshot with the same numbers of nodes n and edges E; grey). Solid line reflects a Poisson distribution. Dashed lines illustrate power laws for comparison.

4 Temporal network datasets

In the main text, we analyze 41 different temporal networks. In Table S1, for each of these networks we provide references and a brief description. We also note that all networks have been made openly available at `github.com/ChrisWLynn/Emergent_scale_free`.

Across all of the temporal networks, in Fig. S2 we illustrate the distributions of different network properties: the total number of nodes N, the total number of edges (that is, the length of the dataset) T, the average degree in our model $\bar{k} = 2E/N$ where $E = 10^3$ (which we note differs from the total average degree $2T/N$ of the entire dataset), the average network size n (that is, the average number of nodes included in each snapshot), the average degree heterogeneity, and the fitted preferential attachment proportion p.

In Fig. S2A, we see that the total number of nodes peaks around $N \approx 10^5$, with a large number of networks just over one million nodes. Meanwhile, the total number of edges peaks near $T \approx 10^6$ (Fig. S2B). Given the numbers of nodes N (Fig. S2A), the average degree peaks near $\bar{k} \approx 0.01$ (Fig. S2C). After grouping the edges into snapshots with $E = 10^3$ edges each, the typical network size is $n \approx 800$ (Fig. S2D), and the average degree heterogeneity peaks strongly between 0.5 and 0.6 (Fig. S2E). Finally, after fitting our model to the degree distribution

Table S1. Descriptions and references for real temporal networks.

Type	Name	Description
Social	Flickr[1,2]	Friendships on Flickr.
	Twitter[3,4]	Retweets on Twitter.
	Facebook[3,5]	Friendships on Facebook.
	Wikipedia (elections)[1,6]	Votes between users in admin elections for English Wikipedia.
	Wikipedia (conflict)[1,7]	Conflicts between users on English Wikipedia.
	Epinions[1,8]	Trust between users on a product rating site.
	Digg[1,9]	Friendships on news aggregator Digg.
	Loans[1,10]	Loans between members of a lending network.
	DBLP[1,11]	Coauthorships from DBLP computer science bibliography.
	Bitcoin (alpha)[1,12]	Trust between users on a Bitcoin trading platform.
	Bitcoin (OTC)[1,12]	Trust between users on a Bitcoin trading platform.
	Coauthors (HEP–Ph)[3,13]	Coauthorships in HEP–Ph section of the arXiv.
	Coauthors (HEP–Th)[3,13]	Coauthorships in HEP–Th section of the arXiv.
Web	Wikipedia (English)[1,14]	Hyperlinks between pages on English Wikipedia.
	Wikipedia (French)[1,15]	Hyperlinks between pages on French Wikipedia.
	Wikipedia (German)[1,15]	Hyperlinks between pages on German Wikipedia.
	Wikipedia (Dutch)[1,15]	Hyperlinks between pages on Dutch Wikipedia.
	Wikipedia (Italian)[1,15]	Hyperlinks between pages on Italian Wikipedia.
	Youtube[3,16]	Hyperlinks between users on Youtube.
	Internet[1,17]	Connections between autonomous systems of the Internet.
Communication	Emails[18]	Emails at a large research institution.
	Wikipedia talk (English)[1,19]	Messages between users on English Wikipedia.
	Wikipedia talk (French)[1,19]	Messages between users on French Wikipedia.
	Wikipedia talk (German)[1,19]	Messages between users on German Wikipedia.
	Wikipedia talk (Spanish)[1,19]	Messages between users on Spanish Wikipedia.
	Wikipedia talk (Dutch)[1,19]	Messages between users on Dutch Wikipedia.
	Wikipedia talk (Italian)[1,19]	Messages between users on Italian Wikipedia.
	Wikipedia talk (Japanese)[1,19]	Messages between users on Japanese Wikipedia.
	Wikipedia talk (Chinese)[1,19]	Messages between users on Chinese Wikipedia.
	Wikipedia talk (Arabic)[1,19]	Messages between users on Arabic Wikipedia.
	Yahoo[3]	Messages between users on Yahoo.
	Enron[1,20]	Emails between Enron employees.
	Slashdot[1,21]	Replies between users.
	Super User[1]	Answers and comments between users on Super User.
	College[22]	Private messages between college students.
	Manufacturing[1,23]	Emails between employees at a manufacturing company.
	Ubuntu[1]	Answers and comments between users on Ask Ubuntu.
	Linux[1,24]	Communication between users in a mailing list.
	Stack Overflow[1]	Answers and comments between users on Stack Overflow.
	Math Overflow[1]	Answers and comments between users on Math Overflow.
Transportation	Flights[25]	Flights between cities.

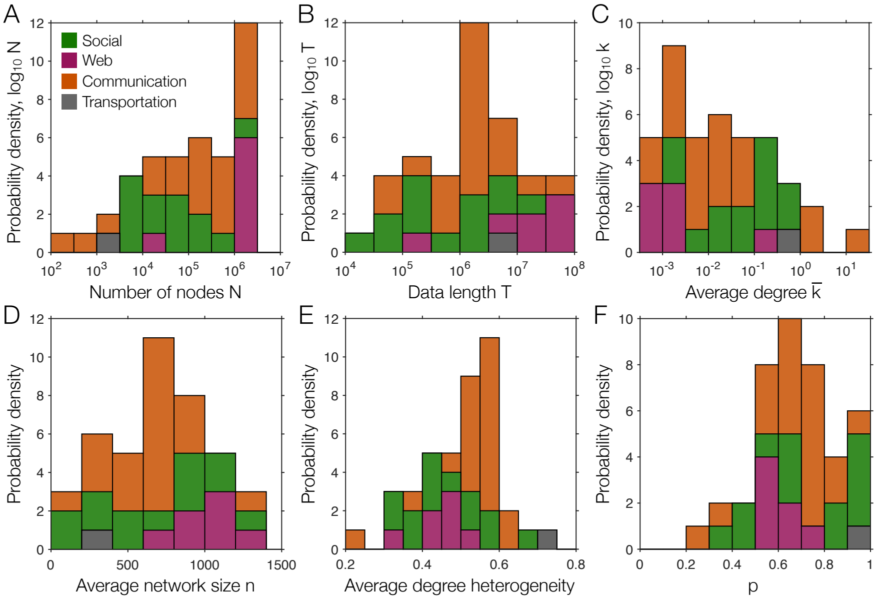

Fig. S2. Distributions of network properties for temporal networks. Distributions over the different temporal networks of the total number of nodes N (*A*), the total number of edges T (*B*), the average degree $\bar{k} = 2E/N$ (*C*), the average network size n (*D*), the average degree heterogeneity (*E*), and the fitted proportion p of preferential attachment (*G*). Panels (*C*-*F*) are computed using snapshots with $E = 10^3$ edges each.

for each temporal network, we find that the proportions p of preferential attachment tend to be slightly greater than half, peaking between 0.6 and 0.7 (Fig. S2F).

5 Dynamics and degree distributions for temporal networks

In the main text, we analyzed the dynamics and degree distributions (Figs. 1 and 4 in the main text) of two real–world temporal networks: a social network of friendships on Flickr and the hyperlinks between pages on English Wikipedia. Here, we present the same analysis for all 41 temporal networks in Table S1.

In Fig. S3, we illustrate the the trajectories of the network sizes n over time. Some networks appear to grow slightly over time (such the Facebook network, the Internet, and English and German Wikipedia), while others appear to slowly decrease in size (such as Epinions and DBLP). However, most networks tend to fluctuate around a steady–state size n. Indeed, by definition each snapshot is limited to $1 \leq n \leq 2E$ nodes (where $E = 10^3$

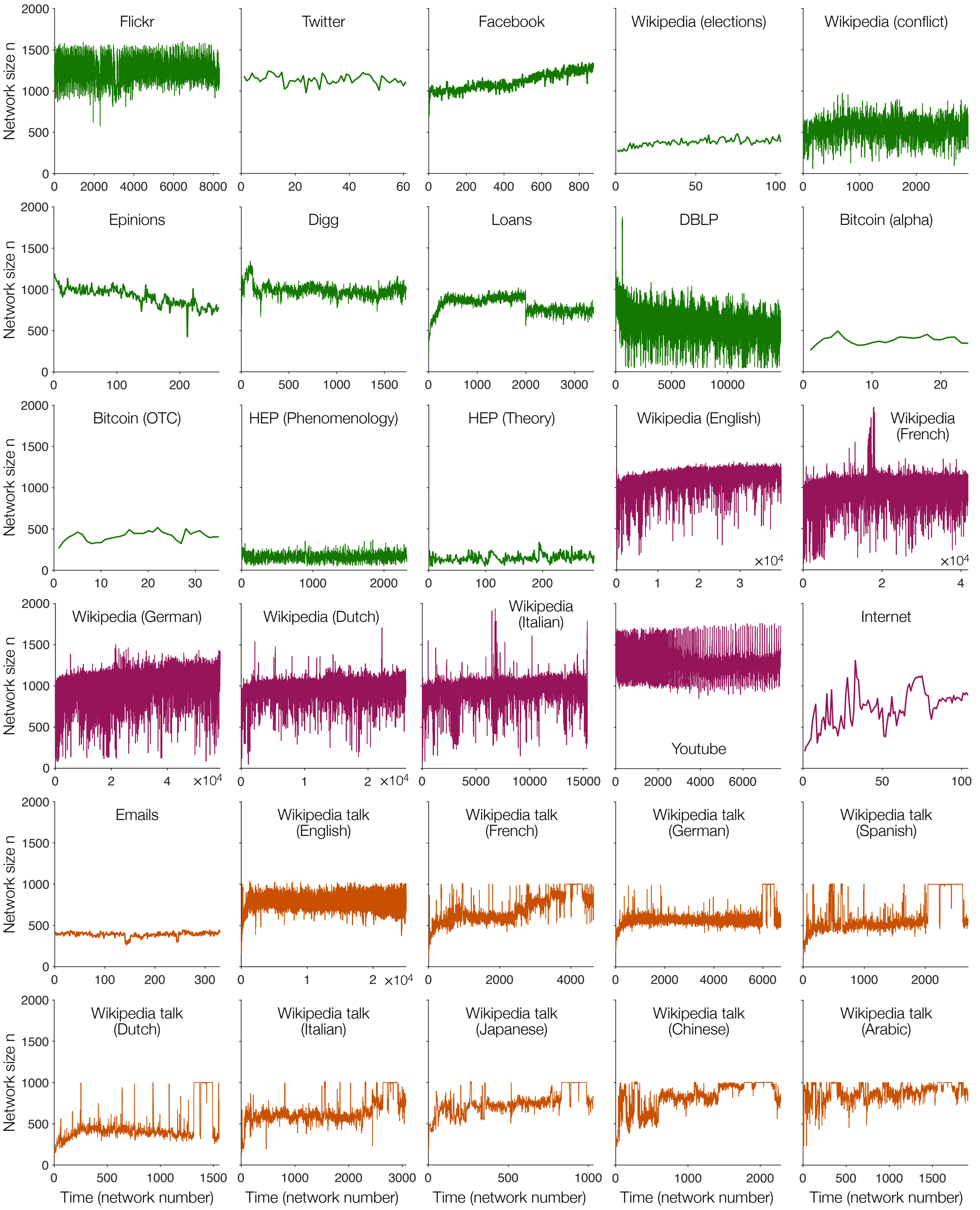
Flickr
Twitter
Facebook
Wikipedia (elections)
Wikipedia (conflict)
Epinions
Digg
Loans
DBLP
Bitcoin (alpha)
Bitcoin (OTC)
HEP (Phenomenology)
HEP (Theory)
Wikipedia (English)
Wikipedia (French)
Wikipedia (German)
Wikipedia (Dutch)
Wikipedia (Italian)
Youtube
Internet
Emails
Wikipedia talk (English)
Wikipedia talk (French)
Wikipedia talk (German)
Wikipedia talk (Spanish)
Wikipedia talk (Dutch)
Wikipedia talk (Italian)
Wikipedia talk (Japanese)
Wikipedia talk (Chinese)
Wikipedia talk (Arabic)
Network size n
Time (network number)
×10^4

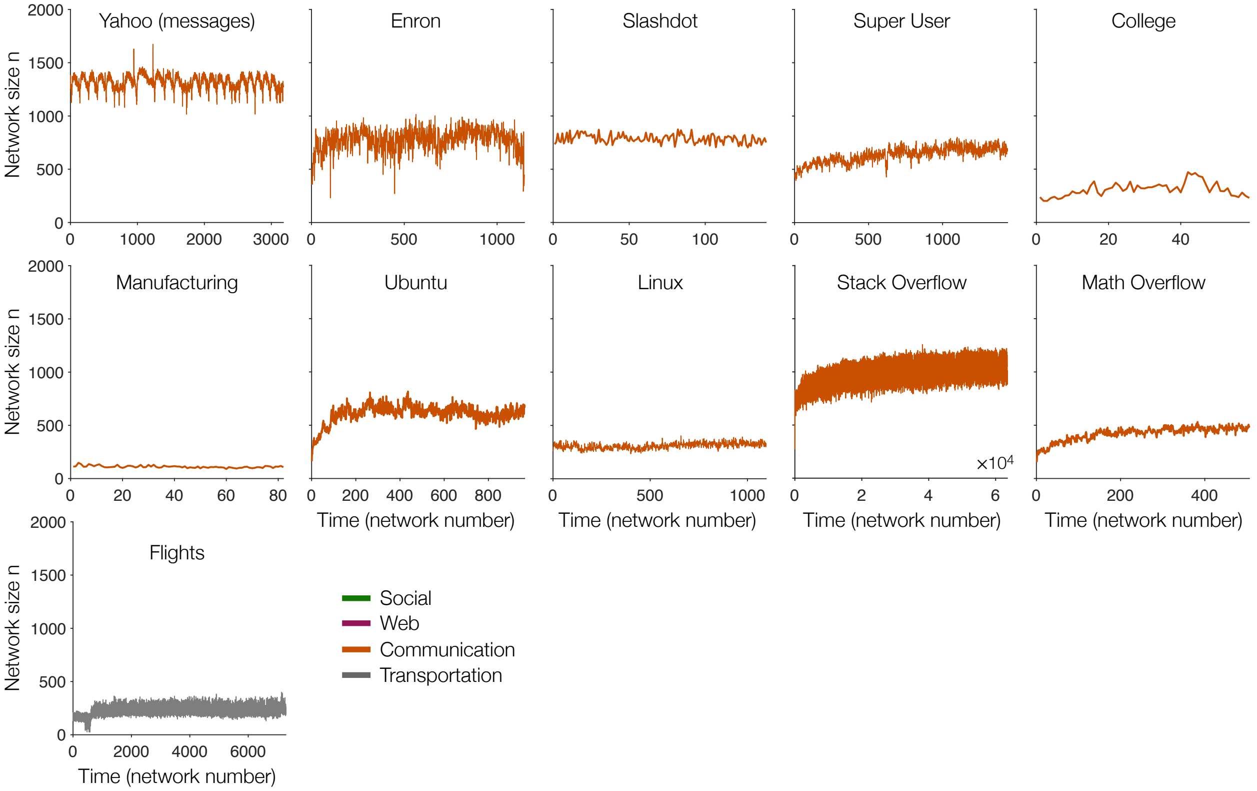

Fig. S3. Evolution of temporal networks. For each of the real temporal networks in Table S1, we plot the number of nodes n in each snapshot as a function of time. Colors indicate network type.

throughout our analysis), and thus the networks cannot grow or shrink without bound.

In Fig. S4, we plot the degree distributions $P(k)$ for each of the temporal networks in Table S1, computed over all snapshots. Despite not growing (Fig. S3), we find that many of the networks exhibit clear scale–free structure, with degree distributions that maintain power–law tails (Fig. S4). We note, however, that not all of the networks display scale–free structure, including coauthorships on the arXiv (HEP–Ph and HEP–Th), manufacturing emails, and interactions on a Linux forum, Stack Overflow, and Math Overflow (Fig. S4). Yet even for these networks, we will see that our model provides a good description of their heavy–tailed degree distributions.

For each of the networks, we fit the analytic degree distribution in Eq. S2. Specifically, we set $\bar{k} = 2E/N$, where $E = 10^3$ and N is the total number of nodes in a given dataset

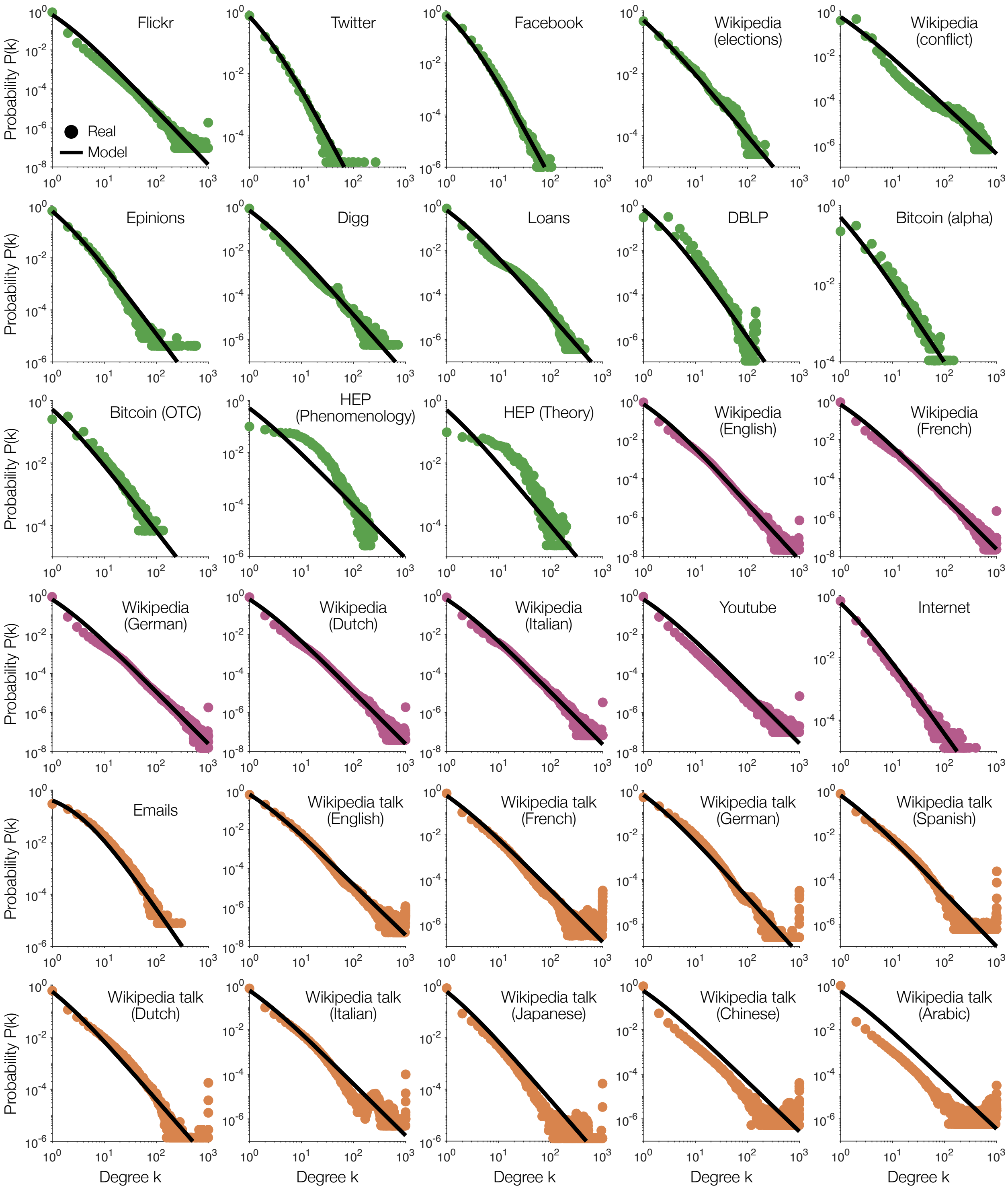
Flickr
Twitter
Facebook
Wikipedia (elections)
Wikipedia (conflict)
Epinions
Digg
Loans
DBLP
Bitcoin (alpha)
Bitcoin (OTC)
HEP (Phenomenology)
HEP (Theory)
Wikipedia (English)
Wikipedia (French)
Wikipedia (German)
Wikipedia (Dutch)
Wikipedia (Italian)
Youtube
Internet
Emails
Wikipedia talk (English)
Wikipedia talk (French)
Wikipedia talk (German)
Wikipedia talk (Spanish)
Wikipedia talk (Dutch)
Wikipedia talk (Italian)
Wikipedia talk (Japanese)
Wikipedia talk (Chinese)
Wikipedia talk (Arabic)
Real
Model
Probability P(k)
Degree k

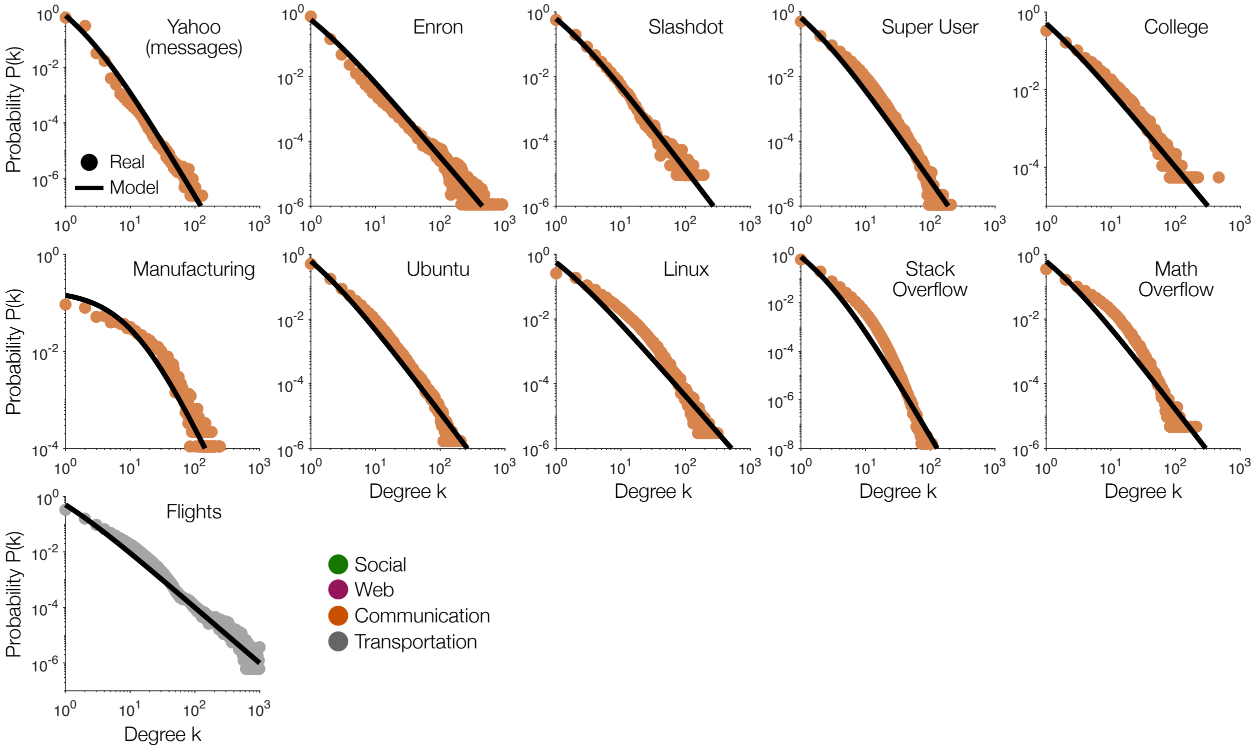

Fig. S4. Degree distributions of temporal networks. For each of the real temporal networks in Table S1, we plot the degree distribution $P(k)$ for snapshots with $E = 10^3$ edges (data points) alongside the one–parameter fits from our model (Eq. S2; lines). Colors indicate network type.

(see Fig. S2C). This leaves one free parameter p, which we fit to minimize the root–mean–squared error between the predicted log probability (Eq. S2) and the observed log probability (see Fig. S2F).

Despite only fitting one free parameter p, our model provides a surprisingly good description for most of the temporal networks (Fig. S4). Our model even provides a close fit for a number of networks that do not exhibit clear scale–free structure, such as the networks of emails between scientists at a research institution (Emails) and emails between employees at a manufacturing company (Manufacturing). We remark that our model appears to provide a poor fit for a number of networks, such as coauthorships on the arXiv (HEP–Ph and HEP–Th) and interactions on a Linux forum, Stack Overflow, and Math Overflow. However, as we will show in the following section, this apparent inaccuracy arises merely from our choice for $\bar{k}$,

and can be easily remedied.

6 Comparison with existing models of scale–free structure

Here we compare our model against previous models for the self–organization of scale–free structure. First, we examine the Barabási–Albert model, which produces scale–free structure with an exponent $\gamma = 3$, but requires the network to constantly grow over time.[26] Second, we present the model of Park, Lai, and Ye, which is capable of generating scale–free structure without growth, but only produces a limited range of exponents $0 \leq \gamma \leq 1$ that do not apply to many real–world networks.[27] Finally, we present our model (described in the main text), which produces a range of scale–free exponents $\gamma \geq 2$ without growth.

6.1 Barabási–Albert model

We begin with the Barabási–Albert model, which has provided the foundation for most existing models of scale–free structure.[26] One begins with an initial network that is fully connected (here we simply begin with a single node). At each step of the dynamics, a new node is added that connects to some number m of the existing nodes (for simplicity, here we use $m = 1$), as illustrated in Fig. S5A. Importantly, the new node connects to existing nodes preferentially; that is, it connects to an existing node i with probability proportional to its degree k_i. Under these dynamics, the network develops a power–law degree distribution $P(k) \sim k^{-\gamma}$ with scale–free exponent $\gamma = 3$ (Fig. S5B). Moreover, subsequent generalizations are capable of generating a wide range of scale–free exponents $\gamma \geq 2$.[28] However, this family of models—indeed, most models of scale–free structure—require networks grow over time (Fig. S5B, *inset*), which (as described in the main text) can fail in some real–world settings.

6.2 Park–Lai–Ye model

In the model of Park, Lai, and Ye, the network is initialized in a ring, with a directed edge from each node i to its neighbor $i + 1$ (Fig. S5C, *left*).[27] At each step of the dynamics, one edge is selected at random, and the directed end is detached. The edge is then reattached preferentially to another node i with probability proportional to $k_i - \alpha$ (Fig. S5C, *right*), where $0 \leq \alpha \leq 1$ (the only parameter in the model) adjusts the baseline attractiveness of nodes.

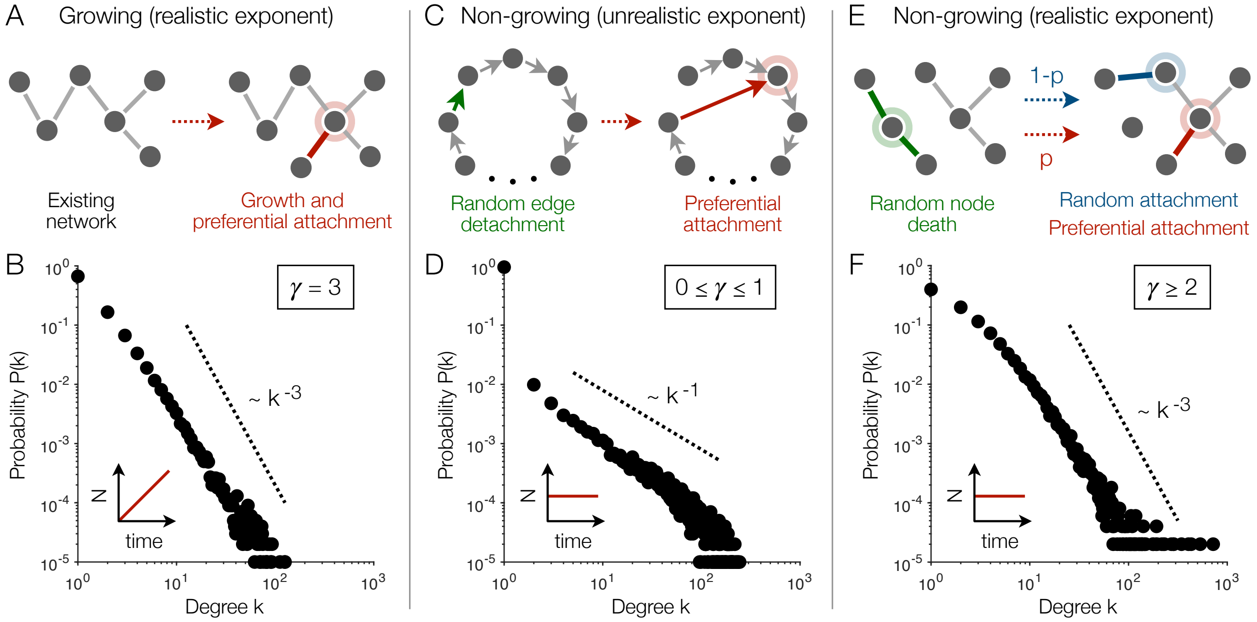

Fig. S5. Comparing models of self–organized scale–free structure. (*A*) The Barabási–Albert model, in which a growing network produces scale–free structure with power–law exponent $\gamma = 3$.[26] (*B*) Degree distribution $P(k)$ of the Barabási–Albert model (with one edge added for each node) after $N = 10^3$ steps of growth. (*C*) The model of Park, Lai, and Ye, in which the number of nodes and edges are held fixed.[27] (*D*) Degree distribution $P(k)$ of the Park–Lai–Ye model for a network with $N = 10^3$ nodes and $\alpha = 0.99$. (*E*) Our model, which does not require growth and produces realistic scale–free exponents $\gamma = 1 + 1/p \geq 2$. (*F*) Degree distribution $P(k)$ of our model for a network with $N = 10^3$ nodes, $E = 10^3$ edges (to match the densities of the other two models), and $p = 0.5$. All degree distributions are calculated over 100 independent network samples.

Under these dynamics, the degree distribution approaches the steady–state form $P(k) \sim k^{-\gamma} e^{-\zeta k}$, which reflects a power law with an exponential cutoff (Fig. S5D). The two parameters are given by $\gamma = \alpha/(2-\alpha)$ and $\zeta = 1 - 1/(2-\alpha)$. In particular, the power–law exponent is constrained to lie in the range $0 \leq \gamma \leq 1$, and therefore cannot generate exponents $\gamma \geq 1$ that describe most scale–free structure in real–world networks. Moreover, due to the exponential cutoff, the model cannot produce distributions with power–law tails. We note that a similar non–growing model was introduced by Xie, Zhou, and Wang that also produces a power–law exponent $\gamma = 1$ with an exponential cutoff.[29] Finally, by adding random weights to the nodes, Park, Lai, and Ye show that their model can generate a power–law exponent $\gamma = 2$;[27]

however, this exponent was only observed numerically (not derived analytically), and relies on the additional assumption of node weights.

6.3 Our model

Here, we present a model for the self–organization of scale–free structure without growth. Our model (i) can be initialized with any network, (ii) is simple enough to allow analytic investigations, and (iii) produces a wide range of power–law exponents $\gamma \geq 2$. As described in the main text, our model is initialized with an arbitrary network of N nodes and E edges (for simplicity, we begin with a random network). At each step of the dynamics, one random node is chosen to detach from the network, such that all of its edges are disconnected (Fig. S5E, *left*). Each disconnected edge is then reattached in the following manner (Fig. S5E, *right*): With probability p, the edge attaches preferentially; that is, it attaches to node i with probability proportional to k_i. Otherwise, with probability $1 - p$, the edge attaches to a random node. As derived in the main text, the network approaches a steady–state degree distribution with a power–law tail $P(k) \sim k^{-\gamma}$ (Fig. S5F). Importantly, the scale–free exponent spans the range of values $\gamma = 1 + 1/p \geq 2$, while the numbers of nodes N and edges E remain constant (Fig. S5F, *inset*).

7 Deviations between real and model networks

In the analytic solution to our model (Eq. S2), we recall that there are two free parameters: the probability of preferential (rather than random) attachment p and the average degree $\bar{k}$. In turn, the average degree is defined by $\bar{k} = 2E/N$, where E is the number of edges in the network at a given time, and N is the total number of nodes that are available to receive a new connection (see Fig. 2 in the main text). When analyzing real temporal networks, we divide the set of edges (i_t, j_t) into snapshots of 10^3 edges each, thus defining $E = 10^3$ in our model. We then choose to set N equal to the total of number nodes that appear at any point in the dataset, which for most networks allows our model to provide an accurate one–parameter fit for p (Fig. S4). However, we note that N in our model (the number of nodes available for connections) need not equal N in the data (the total number of nodes). As we will see, this is

a choice that can lead to inaccurate predictions for a small number of networks.

Consider the network of coauthorships in the HEP–Ph section of the arXiv (Fig. S4). There are a total of 16,595 nodes in the dataset. Yet in our model, when one node attaches to another (that is, when a researcher coauthors a paper with a new collaborator), they are likely to select from a small fraction of the total network; in other words, N in our model is likely to be much smaller than the total number of nodes in the data. Rather than setting these two quantities equal (and then performing a one–parameter fit for p), we can instead do a two–parameter fit for p and $\bar{k}$ (or equivalently, p and N).

As we see in Fig. S6A, after performing a two–parameter fit, our model provides an accurate description of the HEP–Ph degree distribution. Notably, the fitted average degree $\bar{k} = 9.26$ is larger than that in the data $\bar{k} = 0.12$, which corresponds to a much smaller $N = 216$ in our model than then the total number of nodes in the data (16,595). Thus, not only does our model provide an accurate fit, it also predicts that the number of possible new coauthors is around 200, which (as expected) is much smaller than the entire network of researchers in the field.

We can repeat the above analysis for other networks that were poorly fit with one parameter (Fig. S4). After performing two–parameter fits, we confirm that our model provides excellent descriptions of coauthorships in the HEP-Th section of the arXiv (Fig. S6B) and interactions on a Linux forum (Fig. S6C), Stack Overflow (Fig. S6D), and Math Overflow (Fig. S6E). Notably, because each of these networks represents interactions between humans, one might expect the number of possible new connections (N in our model) to be smaller than the total number of people in the dataset. Indeed, for each network, the fitted average degree $\bar{k}$ is consistently larger (and therefore N is consistently smaller) than that observed in the data. Together, these results demonstrate that, by generalizing from one to two free parameters, our model is capable of describing nearly all of the temporal networks in Table S1.

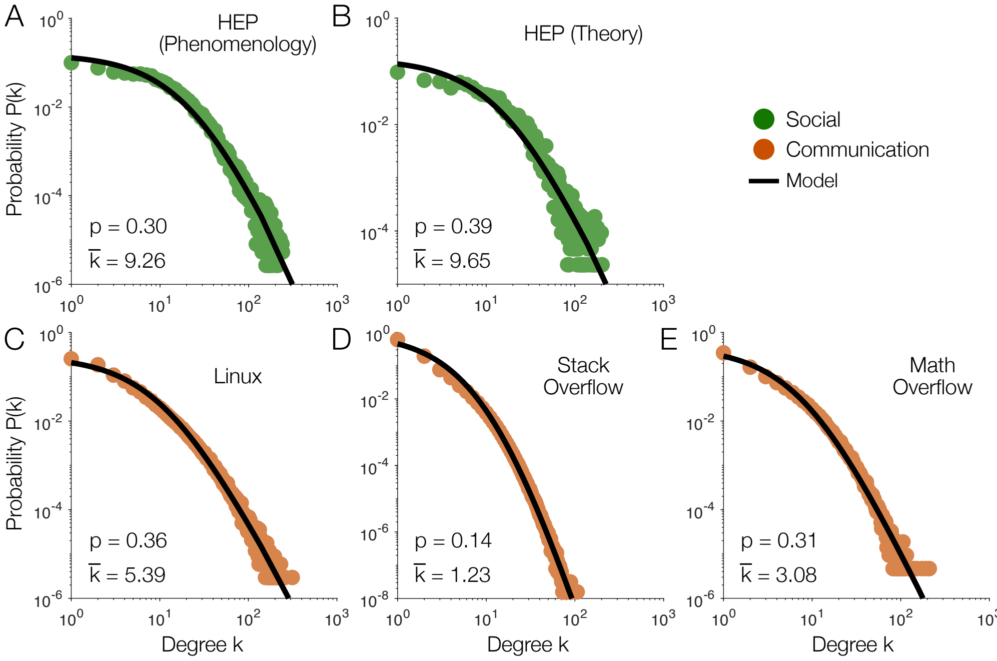

Fig. S6. Two–parameter fits to real degree distributions. We fit both the proportion p of preferential attachment and average degree $\bar{k}$ in our model (Eq. S2) to the degree distributions observed in coauthorship networks on the HEP–Ph and HEP–Th sections of the arXiv [(*A*) and (*B*), respectively] and in networks of interactions on a Linux forum (*C*), Stack Overflow (*D*), and Math Overflow (*E*). Data points depict observed distributions, lines reflect model fits, and colors indicate network type.

8 Properties of temporal network snapshots

In the main text, we showed that our model predicts a specific inverse relationship between the size of a network n and its degree heterogeneity (Fig. 3D in the main text). We then went on to find that the average properties of different real temporal networks displayed a similar pattern (Fig. 4E in the main text). Yet rather than comparing the average properties of different networks, we can instead compare across the many snapshots of a single network.

For each of the temporal networks (Table S1), in Fig. S7 we plot the size n against the degree heterogeneity for different snapshots. For comparison, we also include the predictions of our model while sweeping over p (in the sparse limit $\bar{k} \to 0$; Fig. S7, *solid lines*) and sweeping over $\bar{k}$ (for $p = 0.5$; Fig. S7, *dashed lines*). Notably, every network falls near one or both of these lines. Thus, not only does our simple model capture the relationship between

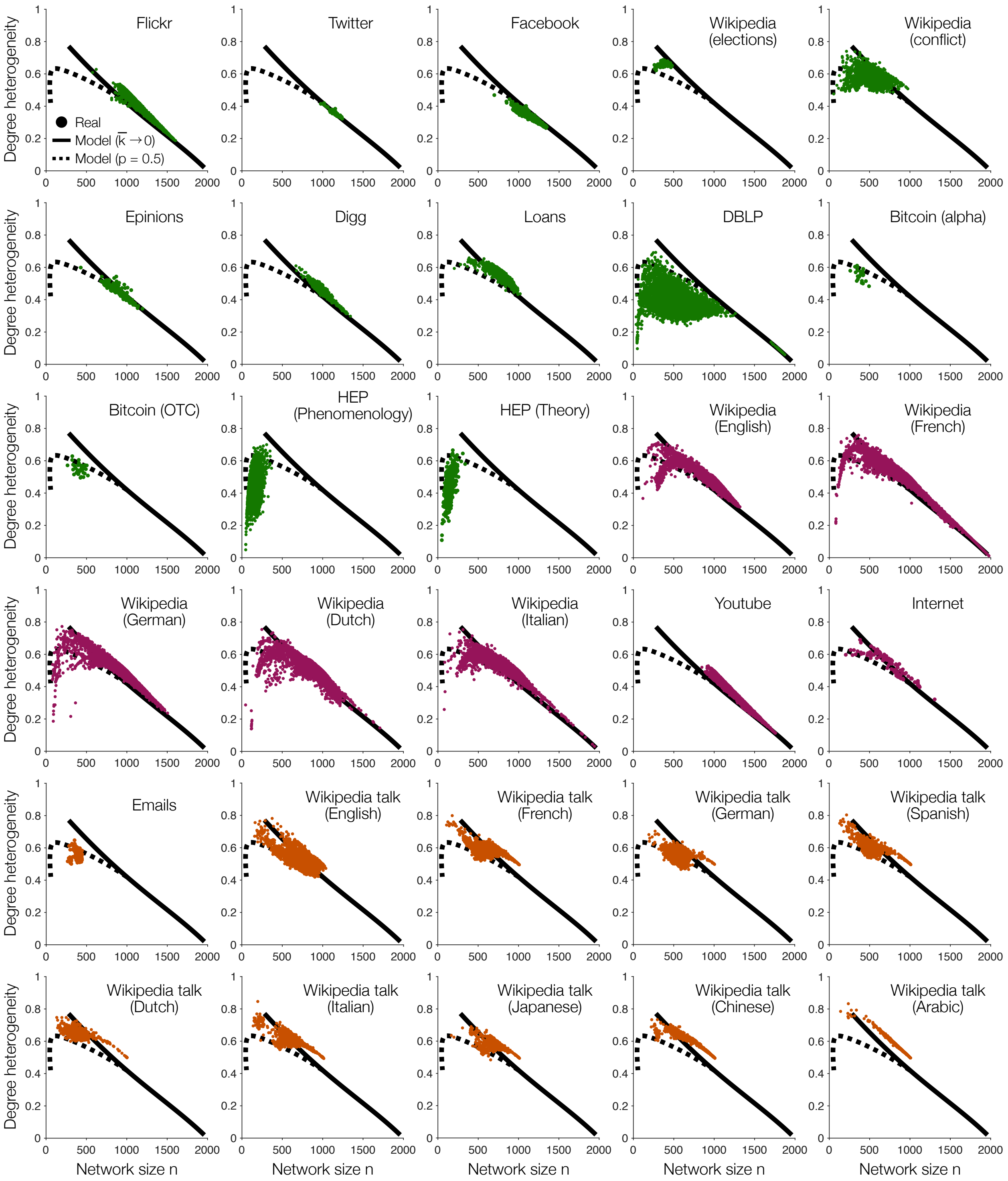

Flickr
Twitter
Facebook
Wikipedia (elections)
Wikipedia (conflict)
Epinions
Digg
Loans
DBLP
Bitcoin (alpha)
Bitcoin (OTC)
HEP (Phenomenology)
HEP (Theory)
Wikipedia (English)
Wikipedia (French)
Wikipedia (German)
Wikipedia (Dutch)
Wikipedia (Italian)
Youtube
Internet
Emails
Wikipedia talk (English)
Wikipedia talk (French)
Wikipedia talk (German)
Wikipedia talk (Spanish)
Wikipedia talk (Dutch)
Wikipedia talk (Italian)
Wikipedia talk (Japanese)
Wikipedia talk (Chinese)
Wikipedia talk (Arabic)
Real
Model ($\bar{k} \to 0$)
Model (p = 0.5)
Degree heterogeneity
Network size n

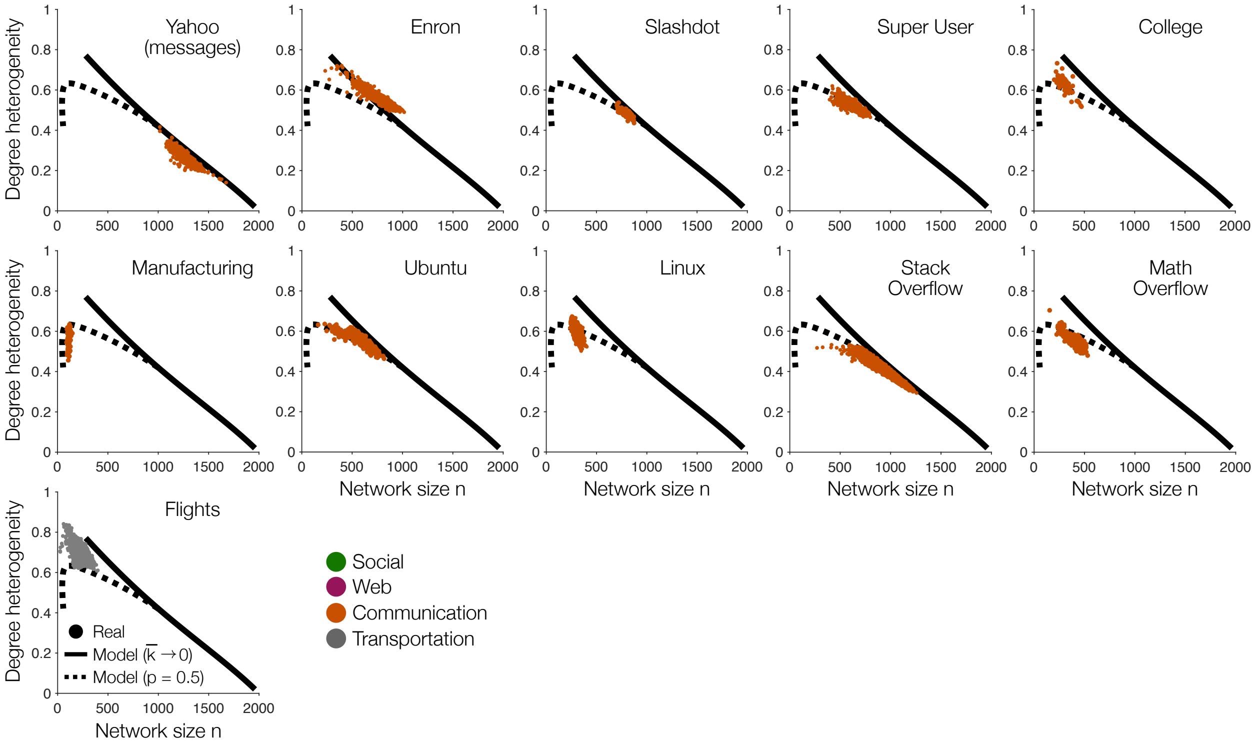

Fig. S7. Properties of temporal network snapshots. For each of the real temporal networks in Table S1, we plot the degree heterogeneity against the size n for the different network snapshots (data points). Lines reflect model predictions while sweeping over p (in the sparse limit $\bar{k} \to 0$; solid lines) and sweeping over $\bar{k}$ (for $p - 0.5$; dashed lines). Colors indicate different network types.

size and heterogeneity across different networks (Fig. 4E in the main text), it also predicts the same relationship across different snapshots of the same network (Fig. S7).

9 Static networks

In the main text, we analyzed the degree distributions of two static networks (Figs. 1A-B and 4A-B in the main text): a network of host–pathogen relationships between animal and plant species and the network of transitions between nouns in Walt Whitman's book *Leaves of Grass*. Here, we analyze a total of 23 static networks, with properties and descriptions listed in Table S2. In particular, we consider unweighted and undirected versions of each network.

In Fig. S8, we plot the degree distributions $P(k)$ for each of the static networks. We find that all of the networks are clearly heavy tailed, with many exhibiting distinct power–law

Table S2. Descriptions, references, and statistics for real static networks.

Type	Name	N	E	p	Description
**Biological**	Host–pathogen[30]	15,024	22,509	1	Host–pathogen interactions.
	Pollination[31]	770	1,202	1	Plant–pollinator interactions.
	Drosophila[32]	1,781	8,911	0.61	Connections between neurons.
	Yeast genetic[33]	688	1,078	1	Genetic interactions.
	E. coli genetic[34]	2,068	4,496	1	Genetic interactions.
	Human protein (Calderwood)[35]	9,094	63,242	0.71	Protein interactions.
	Human protein (Figeys)[36]	2,239	6,432	0.87	Protein interactions.
	Human protein (Wanker)[37]	1,706	3,155	0.98	Protein interactions.
	Yeast protein (Vidal)[38]	2,017	2,704	1	Protein interactions.
	Yeast protein (Oltvai)[39]	1,870	2,203	1	Protein interactions.
	Arabidopsis protein[40]	4,866	10,928	0.98	Protein interactions.
**Information**	Whitman[41]	5,625	19,809	0.79	Noun transitions in *Leaves of Grass*.
	Homer[41]	4,065	28,718	0.66	Noun transitions in the *Iliad*.
	Plato[41]	2,819	12,892	0.69	Noun transitions in the *Republic*.
	Shakespeare[41]	14,262	149,686	0.74	Noun transitions in combined works.
	Cervantes[41]	6,814	50,071	0.71	Noun transitions in *Don Quixote*.
	Austen[41]	2,440	15,610	0.65	Noun transitions in *Pride and Prejudice*.
	Bible[1]	1,773	9,131	0.61	Pronoun co–occurrences.
	Game of Thrones[1]	796	2,823	0.69	Character co–occurrences.
	EPA[3,42]	4,772	8,909	1	Hyperlinks linking to the EPA.
	WebBase[3,43]	16,062	25,593	1	Hyperlinks gathered by web crawler.
	Spam[3,44]	4,767	37,375	0.68	Hyperlinks between spam pages.
**Transportation**	Open Flights[1,45]	2,939	15,677	0.68	Flights between world cities.

tails. We compare each network to our model after performing a one–parameter fit for the preferential attachment probability p. Specifically, we assume that the total number of nodes N and edges E equals the observed numbers in the dataset (see Table S2), and we fit p to minimize the root–mean–square error between the log probability predicted by our model (Eq. S2) and that observed in the data.

Despite only fitting a single parameter p, our model provides a good description for most of the static networks considered (Fig. S8). Our model even provides a close fit for a number of networks that do not exhibit clear scale–free structure, such as the network of neurons in the *Drosophila* brain (Drosophila) and the network of hyperlinks between spam sites (Spam). Thus, our model can be used not only to describe temporally–evolving networks (as in Fig. S4),

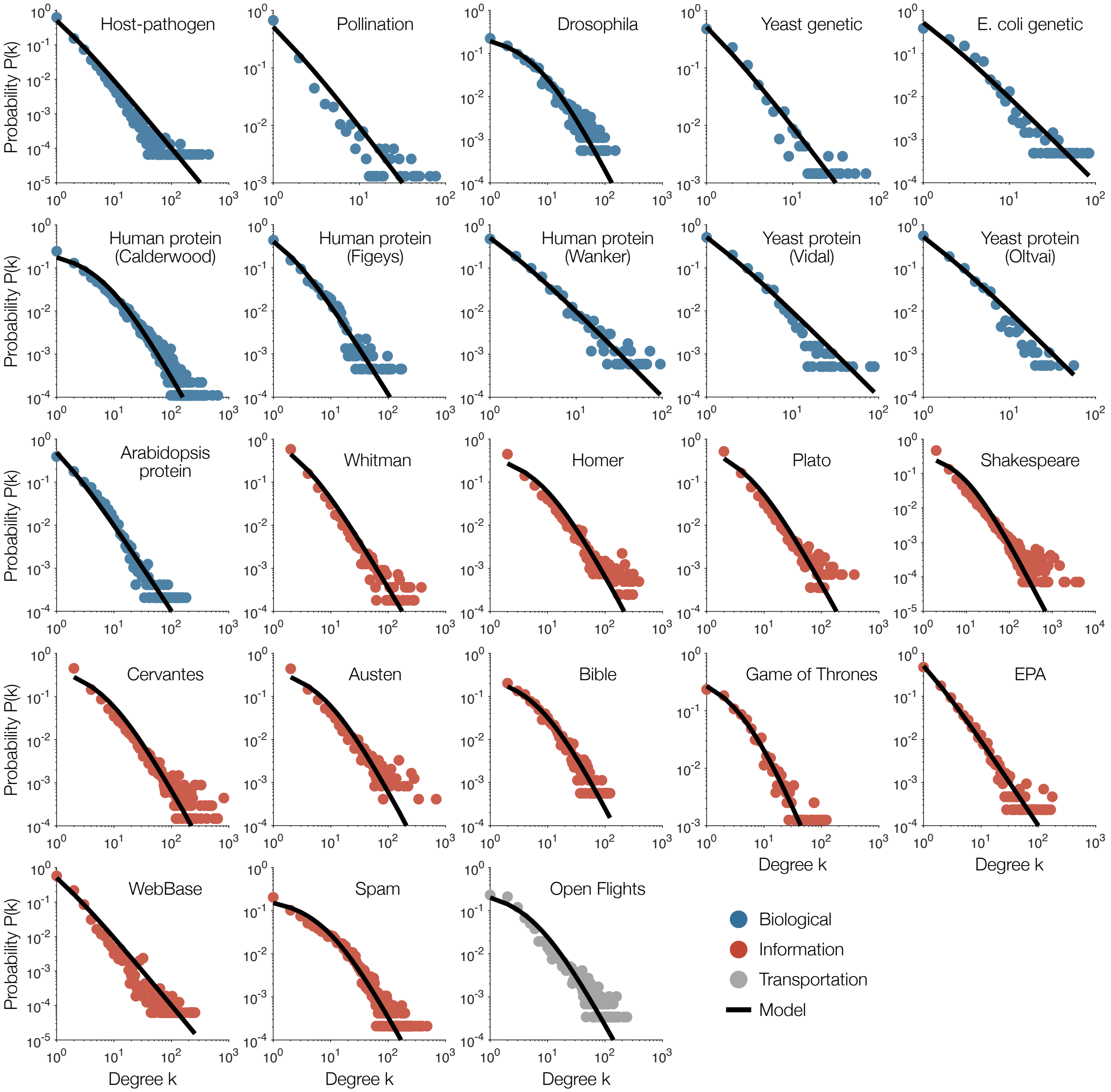

Fig. S8. Degree distributions for real static networks. For each of the real static networks in Table S2, we plot the degree distribution $P(k)$ (data points) alongside the one–parameter fits from our model (Eq. S2; lines). Colors indicate network type.

but also static networks that are not evolving over time.

10 Fractional edge removal

In the main text, we considered a number of extensions to our simple model of emergent scale–free structure (Fig. 5 in the main text). In one of these extensions, rather that removing all of a node's connections when it detaches from the network, we studied the consequences of only removing a fraction f. Now, in addition to p, N, and E, we have an additional free parameter f. For $\bar{k} = 1$, we showed that this fractional edge removal only significantly impacts the degree heterogeneity for $f \lesssim 0.5$ (Fig. 5H-I in the main text). Indeed, in the limit $f \to 1$ we recover the original model.

In Fig. S9, we consider the relative change in the degree heterogeneity between the extension and the original model as we sweep over f and p (for average degrees $\bar{k} = 0.1$, 1, and 10). As expected, the change in degree heterogeneity tends to increase as f decreases (Fig. S9). Notably, we confirm the qualitative result from the main text; namely, that the degree heterogeneity only drops significantly for $f \lesssim 0.5$. Conversely, if nodes lose $f \gtrsim 0.5$ of their connections when they detach from the network, the width of the degree distribution is not significantly impacted (across all values of p and $\bar{k}$).

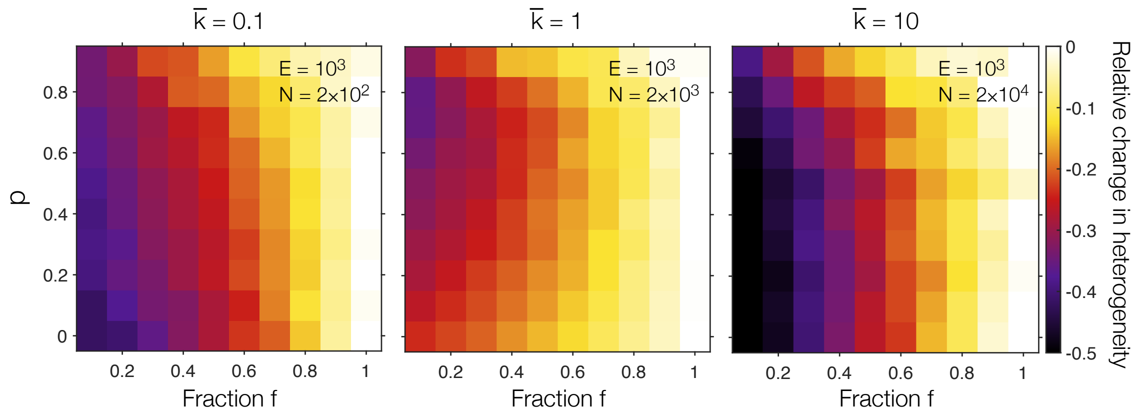

Fig. S9. Fractional edge removal for different average degrees. Relative change in degree heterogeneity after implementing fractional edge removal while sweeping over f and p for average degrees $\bar{k} = 0.1$ (*left*), $\bar{k} = 1$ (*center*), and $\bar{k} = 10$ (*right*). All values are computed using network simulations with $E = 10^3$ edges.

References

1. Kunegis, J. Konect: the koblenz network collection. In *WWW*, 1343–1350 (2013).

2. Mislove, A., Koppula, H. S., Gummadi, K. P., Druschel, P. & Bhattacharjee, B. Growth of the Flickr social network. In *WOSN*, 25–30 (2008).

3. Rossi, R. & Ahmed, N. The network data repository with interactive graph analytics and visualization. In *AAAI* (2015).

4. Conover, M. *et al.* Political polarization on twitter. In *ICWSM* (2011).

5. Viswanath, B., Mislove, A., Cha, M. & Gummadi, K. P. On the evolution of user interaction in facebook. In *WOSN*, 37–42 (2009).

6. Leskovec, J., Huttenlocher, D. & Kleinberg, J. Governance in social media: A case study of the Wikipedia promotion process. In *ICWSM* (2010).

7. Brandes, U., Kenis, P., Lerner, J. & Van Raaij, D. Network analysis of collaboration structure in Wikipedia. In *WWW*, 731–740 (2009).

8. Massa, P. & Avesani, P. Controversial users demand local trust metrics: An experimental study on epinions.com community. In *AAAI*, vol. 1, 121–126 (2005).

9. Hogg, T. & Lerman, K. Social dynamics of Digg. *EPJ Data Sci.* **1**, 1–26 (2012).

10. Redmond, U. & Cunningham, P. A temporal network analysis reveals the unprofitability of arbitrage in the prosper marketplace. *Expert Syst. Appl.* **40**, 3715–3721 (2013).

11. Ley, M. The DBLP computer science bibliography: Evolution, research issues, perspectives. In *SPIRE*, 1–10 (Springer, 2002).

12. Kumar, S., Spezzano, F., Subrahmanian, V. & Faloutsos, C. Edge weight prediction in weighted signed networks. In *ICDM*, 221–230 (IEEE, 2016).

13. Leskovec, J., Kleinberg, J. & Faloutsos, C. Graphs over time: Densification laws, shrinking diameters and possible explanations. In *SIGKDD*, 177–187 (ACM, 2005).

14. Mislove, A. E. *Online social networks: Measurement, analysis, and applications to distributed information systems* (Rice University, 2009).

15. Preusse, J., Kunegis, J., Thimm, M., Staab, S. & Gottron, T. Structural dynamics of knowledge networks. In *ICWSM*, vol. 7, 506–515 (2013).

16. Mislove, A., Marcon, M., Gummadi, K. P., Druschel, P. & Bhattacharjee, B. Measurement and analysis of online social networks. In *ICM*, 29–42 (2007).

17. Zhang, B., Liu, R., Massey, D. & Zhang, L. Collecting the Internet AS-level topology. *SIGCOMM* **35**, 53–61 (2005).

18. Paranjape, A., Benson, A. R. & Leskovec, J. Motifs in temporal networks. In *Proc. ACM WSDM*, 601–610 (ACM, 2017).

19. Sun, J., Kunegis, J. & Staab, S. Predicting user roles in social networks using transfer learning with feature transformation. In *ICDMW*, 128–135 (IEEE, 2016).

20. Klimt, B. & Yang, Y. The Enron corpus: A new dataset for email classification research. In *ECML*, 217–226 (Springer, 2004).

21. Gómez, V., Kaltenbrunner, A. & López, V. Statistical analysis of the social network and discussion threads in slashdot. In *WWW*, 645–654 (2008).

22. Panzarasa, P., Opsahl, T. & Carley, K. M. Patterns and dynamics of users' behavior and interaction: Network analysis of an online community. *J. Assoc. Inf. Sci. Technol.* **60**, 911–932 (2009).

23. Michalski, R., Palus, S. & Kazienko, P. Matching organizational structure and social network extracted from email communication. In *ICBIS*, 197–206 (Springer, 2011).

24. Homscheid, D., Kunegis, J. & Schaarschmidt, M. Private-collective innovation and open source software: Longitudinal insights from linux kernel development. In *I3E*, 299–313 (Springer, 2015).

25. Bureau of Transportation Statistics. Air carrier statistics (2022). Retrieved from: `https://transtats.bts.gov/Tables.asp`.

26. Barabási, A.-L. & Albert, R. Emergence of scaling in random networks. *Science* **286**, 509–512 (1999).

27. Park, K., Lai, Y.-C. & Ye, N. Self-organized scale-free networks. *Phys. Rev. E* **72**, 026131 (2005).

28. Dorogovtsev, S. N. & Mendes, J. F. Evolution of networks. *Adv. Phys.* **51**, 1079–1187 (2002).

29. Xie, Y.-B., Zhou, T. & Wang, B.-H. Scale-free networks without growth. *Physica A* **387**, 1683–1688 (2008).

30. Wardeh, M., Risley, C., McIntyre, M. K., Setzkorn, C. & Baylis, M. Database of host-pathogen and related species interactions, and their global distribution. *Sci. Data* **2**, 1–11 (2015).

31. Kato, M., Makutani, T., Inoue, T. & Itino, T. Insect–flower relationship in the primary beech forest of ashu. *Kyoto: an* (1990).

32. Takemura, S.-y. *et al.* A visual motion detection circuit suggested by *Drosophila* connectomics. *Nature* **500**, 175–181 (2013).

33. Milo, R. *et al.* Network motifs: Simple building blocks of complex networks. *Science* **298**, 824–827 (2002).

34. Salgado, H. *et al.* RegulonDB v8. 0: omics data sets, evolutionary conservation, regulatory phrases, cross-validated gold standards and more. *Nucleic Acids Res.* **41**, D203–D213 (2013).

35. Luck, K. *et al.* A reference map of the human binary protein interactome. *Nature* **580**, 402–408 (2020).

36. Ewing, R. M. *et al.* Large-scale mapping of human protein–protein interactions by mass spectrometry. *Mol. Syst. Biol.* **3**, 89 (2007).

37. Stelzl, U. *et al.* A human protein-protein interaction network: a resource for annotating the proteome. *Cell* **122**, 957–968 (2005).

38. Yu, H. *et al.* High-quality binary protein interaction map of the yeast interactome network. *Science* **322**, 104–110 (2008).

39. Jeong, H., Mason, S. P., Barabási, A.-L. & Oltvai, Z. N. Lethality and centrality in protein networks. *Nature* **411**, 41–42 (2001).

40. Consortium, A. I. M. *et al.* Evidence for network evolution in an Arabidopsis interactome map. *Science* **333**, 601–607 (2011).

41. Lynn, C. W., Papadopoulos, L., Kahn, A. E. & Bassett, D. S. Human information processing in complex networks. *Nat. Phys.* **16**, 965–973 (2020).

42. De Nooy, W., Mrvar, A. & Batagelj, V. *Exploratory social network analysis with Pajek*, vol. 27 (Cambridge University Press, 2011).

43. Boldi, P., Codenotti, B., Santini, M. & Vigna, S. UbiCrawler: A scalable fully distributed web crawler. *Software Pract. Exper.* **34**, 711–726 (2004).

44. Castillo, C., Chellapilla, K. & Denoyer, L. Web spam challenge 2008. In *Proceedings of the 4th International Workshop on Adversarial Information Retrieval on the Web* (2008).

45. Opsahl, T., Agneessens, F. & Skvoretz, J. Node centrality in weighted networks: Generalizing degree and shortest paths. *Soc. networks* **32**, 245–251 (2010).
